# The effect of changing the built environment on physical activity: a quantitative review of the risk of bias in natural experiments

**DOI:** 10.1186/s12966-016-0433-3

**Published:** 2016-10-07

**Authors:** Jack S. Benton, Jamie Anderson, Ruth F. Hunter, David P. French

**Affiliations:** 1 School of Psychological Sciences, University of Manchester, Coupland 1 Building, Oxford Road, Manchester, M13 9PL UK; 2 Department of Architecture, University of Cambridge, Cambridge, UK; 3 UKCRC Centre of Excellence for Public Health (NI)/Centre for Public Health, Queen’s University Belfast, Northern Ireland, UK

**Keywords:** Built environment, Physical activity, Natural experiments, Risk of bias, Review

## Abstract

**Background:**

Evidence regarding the association of the built environment with physical activity is influencing policy recommendations that advocate changing the built environment to increase population-level physical activity. However, to date there has been no rigorous appraisal of the quality of the evidence on the effects of changing the built environment. The aim of this review was to conduct a thorough quantitative appraisal of the risk of bias present in those natural experiments with the strongest experimental designs for assessing the causal effects of the built environment on physical activity.

**Methods:**

Eligible studies had to evaluate the effects of changing the built environment on physical activity, include at least one measurement before and one measurement of physical activity after changes in the environment, and have at least one intervention site and non-intervention comparison site. Given the large number of systematic reviews in this area, studies were identified from three exemplar systematic reviews; these were published in the past five years and were selected to provide a range of different built environment interventions. The risk of bias in these studies was analysed using the Cochrane Risk of Bias Assessment Tool: for Non-Randomized Studies of Interventions (ACROBAT-NRSI).

**Results:**

Twelve eligible natural experiments were identified. Risk of bias assessments were conducted for each physical activity outcome from all studies, resulting in a total of fifteen outcomes being analysed. Intervention sites included parks, urban greenways/trails, bicycle lanes, paths, vacant lots, and a senior citizen’s centre. All outcomes had an overall critical (*n* = 12) or serious (*n* = 3) risk of bias. Domains with the highest risk of bias were confounding (due to inadequate control sites and poor control of confounding variables), measurement of outcomes, and selection of the reported result.

**Conclusions:**

The present review focused on the strongest natural experiments conducted to date. Given this, the failure of existing studies to adequately control for potential sources of bias highlights the need for more rigorous research to underpin policy recommendations for changing the built environment to increase physical activity. Suggestions are proposed for how future natural experiments in this area can be improved.

**Electronic supplementary material:**

The online version of this article (doi:10.1186/s12966-016-0433-3) contains supplementary material, which is available to authorized users.

## Background

Engaging in regular physical activity confers many short- and long-term health benefits for adults [1]. Unfortunately, however, it has been estimated that around 5.3 million global deaths each year are due to insufficient levels of physical activity [2].

The environment in which we live is now widely recognised as a key barrier, or facilitator, to being physically active [3]. One aspect of the environment that is increasingly receiving research attention is the built environment, which refers to physical structures of the environment that have been constructed or modified by people [4]. This includes buildings, open spaces, footpaths, cycle lanes, parks, and trails.

Utilising the built environment as an intervention for improving physical activity offers many advantages. Unlike individual-level approaches, developing a supportive environment has the potential to achieve the biggest reach for long-term, population-wide improvements in physical activity levels [5], and facilitate behaviour change maintenance [6]. Also, physical activity interventions that reach large numbers of people over sustained periods of time are often more cost-effective than individual-level interventions [7].

A large number of studies have found a significant positive association between features of the built environment and physical activity levels [8–10]. Features of the built environment that have been shown to correlate with physical activity levels include mixed land use, population density, street connectivity, and physical infrastructure, including footpaths [9]. However, much of the research to date has relied on cross-sectional studies which cannot show causality.

Natural experiments provide more appropriate study designs for investigating causal effects of the built environment on physical activity. Natural experiments are defined as observational studies that resemble true experiments, but lack random assignment of participants to intervention groups. This is because the intervention is naturally occurring or unplanned and so the researcher does not, and usually cannot, manipulate the intervention exposure or event [11]. Despite this, findings from natural experiments lead to stronger inferences about causality than cross-sectional studies because of the temporal order of changes in environment and behaviour [12]. Due to the difficulties of randomly allocating people to a new neighbourhood or randomising neighbourhoods to be altered using a randomised controlled trial (RCT), natural experiments are therefore most likely the most robust and feasible study design for investigating the causal effects of the built environment on physical activity. Accordingly, many researchers are now increasingly using and recommending natural experiments when evaluating population-level interventions where an RCT is not feasible [11, 13].

There are two important issues that need to be considered when interpreting the results of natural experiments. One of the key issues is that the researcher usually cannot control allocation of participants to intervention and comparison groups. Therefore, differences in outcomes between groups could be explained by other plausible confounding variables, such as demographic features like age or gender [8], and so any observed effect may not be attributable to changes in the built environment if there are not controls for confounders.

Also, well-matched control groups that are unexposed to the intervention are crucial in strengthening the internal validity of natural experiments [11]. Adequately matched control groups reduce the risk of confounding and improve the accuracy of the estimated intervention effect [11]. However, the heterogeneity and complexity of any two neighbourhoods, as well as the various built environment and demographic characteristics that should be matched, makes this a challenging task for researchers.

The issues associated with conducting rigorous natural experiments such as those just outlined increase the potential risk of bias; that is, the risk of systematic errors in estimations of the causal effect due to the design, conduct, analyses and reporting within a study [14]. Despite this, according to a review of reviews in this research area [15], only a minority of systematic reviews followed the PRISMA (Preferred Reporting Items for Systematic reviews and Meta-Analyses) guidelines [16] to assess the methodological quality of included studies. This is a major concern, particularly as non-randomised studies such as natural experiments are more prone to bias than RCTs [17].

Although some reviews have attempted to assess the methodological quality of natural experiments in this area [18–20], these attempts have not been optimal. For example, Hunter et al. [19] recently appraised eleven natural experiments and one RCT using the Cochrane Risk of Bias tool [14], which was specifically designed for randomised trials. This tool is inappropriate for natural experiments because it includes criteria irrelevant to natural experiments, such as allocation sequence and allocation concealment, and omits key criteria relevant to natural experiments, such as bias in measurement of interventions [21].

Previous reviews that have included evidence from the limited number of natural experiments tend to conclude that built environment interventions lead to increases in physical activity levels, but the effect sizes are generally more modest than single cross-sectional studies [18–20]. Nevertheless, several researchers have proposed that sufficient evidence exists to recommend built environment interventions for the purposes of increasing physical activity, despite the small number of natural experiments and absence of an adequate assessment of potential bias in these studies [18, 22–25]. As these proposals are now starting to be reflected in policy guidelines for physical activity worldwide [26–30], it is now essential to assess the quality of the evidence.

The aim of the present review was to conduct a thorough quantitative appraisal of the risk of bias present in those natural experiments which had the strongest experimental designs for assessing the causal effects of the built environment on physical activity. Eligible studies had to evaluate the effects of changing the built environment on physical activity, include at least one measurement before and one measurement of physical activity after changes in the environment, and have at least one intervention site and non-intervention comparison site.

Given that at least 31 systematic reviews have already examined the built environment-physical activity relationship [15], a new systematic search of the literature was deemed redundant. Instead, studies for the present review were obtained from three recent peer-reviewed systematic reviews that covered different types of built environment interventions [10, 19, 20].

## Methods

### Inclusion criteria

Studies were included only if they: (i) were included in one of three existing exemplar systematic reviews [10, 19, 20]; (ii) were natural experiments; that is, evaluated interventions that involved a change to the built environment and researchers did not control intervention allocation; (iii) had physical activity as an outcome, including overall physical activity, walking, cycling, active travel, or pedestrian counts; (iv) had outcomes that were taken before and after environmental change; (v) had at least one control/comparison group; (vi) included adults; and (vii) were published in English.

Evaluations of the following interventions were excluded: (i) indoor environments; (ii) changes to the socioeconomic or political environment; and (iii) residential relocation.

The three exemplar peer-reviewed systematic reviews were chosen from the plethora of existing reviews for five key reasons:

1. They all included natural experiments evaluating changing the built environment on physical activity;
2. Each review included different types of interventions, from urban green space to public buildings, thus providing a complementary breadth of coverage of research in this area;
3. A diverse range of nine unique databases were searched;
4. The reviews were published within the past five years;
5. The reviews were transparent in their reporting, which has been an issue with many previous reviews in this area [15].

All primary studies in the three exemplar systematic reviews were assembled and duplicates removed. The first author and a second coder then independently screened the full texts of these studies to select those that met the inclusion criteria. The agreement between coders was very good with agreement on 94 % of studies (κ = 0.81) [31]. Any differences between coders were resolved by discussion.

### Critical appraisal tool: ACROBAT-NRSI and adaptations

Risk of bias was assessed using A Cochrane Risk of Bias Assessment Tool: for Non-Randomized Studies of Interventions (ACROBAT-NRSI) [21]. This tool was chosen because it is specifically designed for non-randomised studies [32].

The ACROBAT-NRSI includes seven domains of bias, which are split into three sections: pre-intervention, at-intervention and post-intervention. A risk of bias judgement is required in all domains for each individual outcome in a study, from which an overall risk of bias judgement is then made. Risk of bias judgements can be scored as ‘low’, ‘moderate’, ‘serious’ or ‘critical’, as well as a ‘no information’ option for when there is insufficient information to make a judgement.

Each domain of bias in the ACROBAT-NRSI contains signalling questions; these are factual questions that provide an evidential basis for risk of bias judgements. Response options include ‘yes’, ‘probably yes’, ‘probably no’, ‘no’, and ‘no information’, whereby ‘yes’ indicates a low risk of bias. An example of a signalling question within the ‘bias in measurement of outcomes’ domain is: ‘was the outcome measure objective?’ ([21]: p. 52), so a response of ‘yes’ indicates a low risk of bias. All signalling questions are structured in this manner.

The ACROBAT-NRSI states that if an outcome is at a particular level of risk of bias for any of the seven domains, then the overall risk of bias will be at least this severe. For example, a serious risk of bias in any domain will result in at least an overall serious risk of bias, regardless of the domain that contains this bias. The ACROBAT-NRSI also proposes that risk of bias is additive, so that moderate or serious risks of bias in multiple domains leads to a higher overall risk of bias; however, there was no specified threshold for this additive risk. Therefore, to maintain consistency throughout the analysis, if an outcome has a particular risk of bias (e.g. “serious”) in at least four domains, then this outcome has an overall risk of bias of the next highest level (e.g. “critical”).

### Adapting the ACROBAT-NRSI

Although the ACROBAT-NRSI was designed for use with natural experiments, it was adapted for the present review for two key reasons. Firstly, the tool did not consider many of the important aspects of research specifically relevant to this field, such as control site selection and measuring exposure to the intervention. Secondly, the ACROBAT-NRSI only focuses on studies’ internal validity, i.e. the extent to which evidence of causality can be established from a study’s findings [33]. This is only the second of four cumulative validity questions that need to be considered when evaluating the overall validity of a study [33].

The ACROBAT-NRSI was adapted to include two other types of validity: statistical conclusion validity (the first cumulative validity question), which looks at the degree to which estimations about the relationship between two sample variables is true of the population, [33], and construct validity (the third cumulative validity question), which extends beyond the causal relationship and examines whether the constructs being investigated actually reflect the constructs of interest [33]. A further fourth type of validity, external validity, referring to the generalisability of causal inferences, was not considered for this review because the aim was to establish whether a causal relationship exists between the built environment and physical activity.

As recommended by the ACROBAT-NRSI, a list of the critically important confounding domains in this research area was identified using scoping reviews of the literature (see Table 1). Additional signalling questions were created for all the methodological features that need to be considered when evaluating natural experiments in this area. All signalling questions were based on relevant guidance and evidence; which included Medical Research Council (MRC) guidance on how to conduct natural experiments [11], UK National Institute for Health and Care Excellence (NICE) guidance on the physical environment and physical activity [29, 34], existing reviews in this area (e.g., [35]), and other relevant literature (e.g., [36]) (Additional file 1 contains a full description).

**Table 1:** Summary of the seven bias domains and types of signalling questions added to the ACROBAT-NRSI

Bias domain	Definition	Types of signalling questions added to the ACROBAT-NRSI
1) Bias due to confounding	Confounding occurs when one or more variables also explain the observed relationship between exposure and outcome.	The following four critically important confounding domains were identified: (1) baseline outcome measurements; (2) baseline demographic characteristics (including age and gender as a minimum standard); (3) any unusual events; and (4) socioeconomic or political influences. Following this, a number of signalling questions were also added to this bias domain concerning the control site; including how well the control and intervention site were matched in terms of built environment features and population demographics, whether there were multiple control sites, and whether any significant changes occurred to the control site during the study period.
2) Bias in selection of participants into the study	This bias domain refers to the exclusion of eligible participants that biases the outcome.	Signalling questions were added to determine whether a fully justified sample size calculation was carried out, and whether both the sampling criteria and the sample were clearly described.
3) Bias in measurement of interventions	Bias in this domain occurs when intervention status is misclassified; that is, when errors in measuring participants exposure to the intervention biases the estimated effect of the intervention.	Signalling questions were added concerning whether the selection of the sampling site was appropriate and justified, and also whether the intervention was clearly reported in terms of what was modified, where the intervention was implemented, and how long it took to construct the intervention.
4) Bias due to departures from intended interventions	This bias domain refers to systematic differences between intervention and control groups due to departures from the intended intervention.	Signalling questions were added to consider whether any delays or changes in intervention construction impacted upon the study, and whether individual-level intervention exposure was measured.
5) Bias due to missing data	Studies that have missing data increase the risk of selection bias, thus resulting in a misrepresented sample.	Signalling questions were added for the response rates at baseline, follow-up, and the overall response rate.
6) Bias in measurement of outcomes	Bias can occur when there are errors in measuring outcomes of the intervention.	Additional signalling questions related to whether outcome measures were clearly described, valid and reliable, timing of measurements, whether there were multiple follow-up time points, and potential performance biases due to participants’ awareness of the study.
7) Bias in selection of the reported result	This domain refers to the selective reporting of fully reported results.	Signalling questions added to this section focused on whether a pre-registered study protocol was published specifying the objectives and methods of the study.

Forty-nine unique signalling questions were added to the ACROBAT-NRSI. An overview of all signalling questions added to the ACROBAT-NSRI can be found in Additional file 2 and examples are given in Table 5. These additional signalling questions were mapped onto the ACROBAT-NRSI under the relevant domains of bias and were structured in the same manner as the original tool. The only signalling questions removed were those specifically for case-control studies and one signalling question relating to implementation failure (intervention fidelity), as these were irrelevant to studies in the present review.

Following this, an iterative review and refinement process was carried out, including refinements by the third and fourth authors. An accompanying guidance document was developed which contained notes and criteria to provide decision rules for using the signalling questions when judging the risk of bias in each bias domain (Additional file 3).

### Overview of signalling questions in the adapted ACROBAT-NRSI

A total of 79 signalling questions were used covering seven bias domains from the ACROBAT-NRSI shown in Table 1.

### Risk of bias assessment

Initially, to ensure that the ACROBAT-NRSI operated efficiently and to improve inter-rater agreement, three authors independently assessed the risk of bias in four included studies (33 %) that were randomly selected. Following minor modifications, the first and second authors independently assessed the remaining studies using the final version of the adapted ACROBAT-NRSI (Additional file 4). Any differences between assessors were resolved by discussion. The first author reassessed the first four randomly selected studies using the final version of the adapted ACROBAT-NRSI.

### Analysis

Once all risk of bias assessments were completed, frequencies of each risk of bias judgement were counted in all bias domains to examine which outcomes had the highest risk of bias. Frequencies were also calculated across all seven bias domains to establish which domains produced the highest risk of bias for all outcomes.

## Results

There were a total of 82 studies included in the three exemplar systematic reviews. Ten duplicate studies were found, leaving a total of 72 unique studies. Twelve studies met the inclusion criteria and were thus included in this review (see Table 2).

**Table 2:** Summary of the key characteristics of included studies

Author (date)	Study location	Research design	Type of intervention (total cost)	Physical activity outcomes^a^ (level of data)	Sample size	Number/type of control sites
Branas et al. [37]	US	Repeated cross-sectional	Greening of 4,436 abandoned vacant lots over 725,000 m^2^(cost not reported)	Self-report survey (individual-level)	No exact count provided	13,308 matched control lots at a ratio of 3:1 per treated lot
Cohen et al. [38]	US	Mixed	5 parks, ranging from 3.4 to 16 acres, underwent major improvements (Over $1 million budget per park)	1) Systematic observation using SOPARC (population-level) 2) Self-report household interviews (indivudal-level) 3) Self-report intercept interviews (individual-level)	1) 3,500 park users 2) 1,480 park users 3) 1,387 household residents	5 matched control parks
Cohen et al. [39]	US	Repeated cross-sectional	A skate park ($3.5 million) and a senior citizen’s centre ($3.3 million) had major renovations	Systematic observation using SOPARC (population-level)	Senior centre: 2,188 users; Skate park: no exact count provided	1 control site per intervention; one skate park and one senior centre
Cohen et al. [40]	US	Repeated cross-sectional	12 parks, ranging from 0.5 to 46 acres, had “Family Fitness” Zones (outdoor gyms) installed (average of $45,000 per park)	1) Systematic observation using SOPARC (population-level) 2) Self-report intercept interviews (individual-level)	1) 9,476 park users 2) 2,636 interviews	10 matched control parks
Fitzhugh et al. [41]	US	Repeated cross-sectional	A 2.9-mile, 8-foot wide urban greenway/trail was retrofıtted in a neighbourhood ($2.1 million)	Systematic observation (population-level)	No exact count provided	2 matched control neighbourhoods
Gustat et al. [42]	US	Repeated cross-sectional	A 6-block walking path and a school playground were installed (cost not reported)	1) Self-report survey (individual-level) 2) Systematic observation using SOPARC/SOPLAY (population-level)	1) 1,191 interviews 2) No exact count provided	2 matched control neighbourhoods
Krizek et al. [43]	US	Repeated cross-sectional	Installation of bicycle lanes and off-street bicycle paths (cost not reported)	Self-report census data (indivudal-level)	No exact count provided	1 buffer zone based on distance from intervention facilities
Merom et al. [44]	Australia	Mixed	Construction of a Rail Trail and a local promotional campaign to raise awareness of the facility (cost not reported)	1) Self-report survey (individual-level) 2) Systematic observation (population-level)	1) 450 households at follow-up 2) No exact count provided	1 outer area located 1.5 to 5 km from the Rail Trail
Parker et al. [45]	US	Repeated cross-sectional	A 1-mile, 5-foot wide bike lane was constructed (cost not reported)	Systematic observation (population-level)	No exact count provided	2 adjacent streets
Tester and Baker [46]	US	Repeated cross-sectional	2 public parks underwent playfield renovations and staff development programs ($5.5 million)	Systematic observation using SOPARC (population-level)	4,889 park visitors	1 matched control park
Veitch et al. [47]	Australia	Repeated cross-sectional	A park (size: 25,200 m^2^) was refurbished (cost not reported)	Systematic observation using SOPARC (population-level)	2,050 park users	1 matched control park (size: 10,000 m^2^)
West and Shores [48]	US	Within-person longitudinal	5 miles of greenway added to an existing greenway (cost not reported)	Self-report survey (individual-level)	166 residents	1 buffer zone based on distance from greenway

^a^SOPARC (System for Observing Play and Recreation in Communities) [49] is a validated instrument for measuring physical activity using systematic observation in community settings; SOPLAY (System for Observing Play and Leisure Activity in Youth) [50] is a validated instrument for measuring physical activity using systematic observation in free play settings (e.g., during lunchtime at school)

The remaining 60 studies were excluded because they used a cross-sectional design (*n* = 16); the researchers evaluated residential relocation (*n* = 8); physical activity was not included as an outcome (*n* = 20); there was no control/comparison group (*n* = 9); there was no pre-post test for both intervention and control groups (*n* = 1); the study evaluated whether participants exposed to the intervention behaved differently compared to unexposed participants, rather than evaluating whether physical activity levels subsequently changed following the built environment intervention (*n* = 1); there was no change in the built environment (*n* = 2); only children or adolescents were recruited (*n* = 3).

### Study characteristics

A summary of the key characteristics and results of all 12 included studies is presented in Table 2. There was much variation in research design, location, intervention type, outcome measures, sample sizes and number/type of control sites between studies (see further details in Additional file 5).

### Risk of bias

As recommended by the ACROBAT-NRSI, separate risk of bias assessments were conducted for each outcome in studies with multiple outcomes. Therefore, risk of bias was assessed in terms of individual outcomes rather than individual studies.

There were a total of 17 unique physical activity outcomes in the 12 included studies. For one study that had two outcomes [44], one observational outcome was excluded because there was no control site for this specific outcome. In another study [38], household interviews and intercept surveys were treated as one outcome because the researchers combined these outcomes in their analysis. A total of *n* = 15 outcomes from *k* = 12 studies underwent a risk of bias assessment.

The two assessors gave the same judgement in 76 % of bias domains for 10 outcomes across eight studies (the four remaining studies were used in the piloting of the ACROBAT-NRSI and were thus excluded from the inter-rater reliability assessment). The inter-rater reliability of agreement across the seven domains of bias was therefore “good” (κ = 0.63) according to conventional criteria [31].

#### Risk of bias in all outcomes

Most outcomes had an overall critical risk of bias (*n* = 12), whilst the remaining outcomes had an overall serious risk of bias (*n* = 3). The outcome with the highest risk of bias in this review was Merom et al. [44], as their self-report outcome had a critical risk of bias in two domains (see Table 3). The systematic observation outcome of Veitch et al. [47] had the lowest risk of bias, as only two domains had a serious risk of bias (see Table 3).

**Table 3:** Summary of risk of bias judgements for included outcomes

Author (date)	Outcome	Pre-intervention		At-intervention	Post-intervention				Overall bias^a^
		Bias due to confounding	Bias in selection of participants into the study	Bias in measurement of interventions	Bias due to departures from intended interventions	Bias due to missing data	Bias in measurement of outcomes	Bias in selection of the reported result	
Branas et al. [37]	Self-report	Serious	Serious	Moderate	Serious	Serious	Serious	Serious	Critical
Cohen et al. [38]	1) Systematic observation	Serious	Serious	Moderate	Low	Low	Serious	Serious	Critical
	2) Self-report	Serious	Moderate	Moderate	Moderate	Moderate	Serious	Serious	Serious
Cohen et al. [39]	Systematic observation	Serious	Serious	Moderate	Low	Low	Serious	Serious	Critical
Cohen et al. [40]	1) Systematic observation	Serious	Moderate	Low	Low	Low	Critical	Serious	Critical
	2) Self-report	Serious	Moderate	Low	Low	Moderate	Critical	Serious	Critical
Fitzhugh et al. [41]	Systematic observation	Serious	Serious	Serious	Low	Low	Serious	Serious	Critical
Gustat et al. [42]	1) Self-report	Serious	Moderate	Low	Moderate	Low	Serious	Serious	Serious
	2) Systematic observation	Serious	Serious	Low	Low	Low	Serious	Serious	Critical
Krizek et al. [43]	Self-report	Critical	Serious	Serious	Serious	No information	Serious	Serious	Critical
Merom et al. [44]	Self-report	Serious	Critical	Serious	Moderate	Moderate	Critical	Serious	Critical
Parker et al. [45]	Systematic observation	Serious	Serious	Serious	Low	Low	Serious	Serious	Critical
Tester and Baker [46]	Systematic observation	Serious	Moderate	Low	Serious	Moderate	Serious	Serious	Critical
Veitch et al. [47]	Systematic observation	Serious	Moderate	Low	Low	Low	Low	Serious	Serious
West and Shores [48]	Self-report	Serious	Moderate	Serious	Serious	Serious	Serious	Serious	Critical

^a^If an outcome is at a particular level of risk of bias for any of the seven domains (e.g. serious), then the overall risk of bias will be at least this severe (e.g., serious). If an outcome has moderate or serious risks of bias in four or more domains, then the outcome has an overall serious or critical risk of bias judgement respectively

#### Risk of bias across each domain

The majority of outcomes had a serious risk of bias due to: confounding (*n* = 14), measurement of outcomes (*n* = 11), and selection of the reported result (*n* = 15) (see Table 4). Only a minority of outcomes had a serious risk of bias due to missing data (n = 2) (see Table 4). Other domains that had low numbers of outcomes with a serious risk of bias were bias in measurement of interventions (*n* = 5) and bias due to departures from intended interventions (*n* = 4) (see Table 4). Some outcomes had a critical risk of bias due to: confounding (*n* = 1), selection of participants into the study (*n* = 1), and measurement of outcomes (*n* = 3). Table 5 displays the signalling questions that contributed most to the high risk of bias in each domain.

**Table 4:** Frequency of risk of bias judgements in each bias domain across included outcomes

Risk of bias judgement	Pre-intervention		At-intervention	Post-intervention				Overall bias
	Bias due to confounding	Bias in selection of participants into the study	Bias in measurement of interventions	Bias due to departures from intended interventions	Bias due to missing data	Bias in measurement of outcomes	Bias in selection of the reported result	
Low	-	-	6	8	8	1	-	-
Moderate	-	7	4	3	4	-	-	-
Serious	14	7	5	4	2	11	15	3
Critical	1	1	-	-	-	3	-	12
No information	-	-	-	-	1	-	-	-

**Table 5:** Descriptive statistics of signalling questions that contributed most to the high risk of bias

Bias domain	Signalling question	Judgement	Frequency of eligible outcomes	Percentage of eligible outcomes^a^
Bias due to confounding	Did the authors use an appropriate analysis method that adjusted for all the critically important confounding domains?	Yes	0	0 %
		No	15	100 %
	Critically important confounding domains not controlled for			
	Differences in baseline outcome measurements	-	10	66.6 %
	Differences in baseline demographic characteristics	-	9	60 %
	Any unusual events	-	4	27 %
	Socioeconomic or political influences	-	2	13 %
	What variables were used to match intervention and control sites?			
	Demographic variables (e.g., ethnicity, income, education)	-	5	62.5 %
	Features, facilities or amenities	-	5	62.5 %
	Size	-	2	25 % 12.5 %
	Land use	-	1	
	Same neighbourhood	-	1	12.5 %
	Is the control site well matched to the intervention site?	Yes	4	26.7 %
		No No information	9 2	60 % 13.3 %
	Were there multiple control sites?	Yes	9	60 %
		No	6	40 %
Bias in selection of participants into the study	Is there a fully justified sample size calculation?	Yes	0	0 %
		No	15	100 %
	Is there a clear and sufficient description of the sample?	Yes	5	33 %
		No	7	47 %
		Not applicable^b^	3	20 %
Bias in measurement of interventions	Did the authors describe…			
	… what was modified in the intervention?	Yes	15	100 %
		No	0	0 %
	… where the intervention was implemented?	Yes	9	60 %
		No	6	40 %
	… how long it took to construct the intervention?	Yes	4	26.7 %
		No	8	53.3 %
		No (and potential overlap with intervention construction)	3	20 %
Bias due to departures from intended interventions	Was individual-level intervention exposure measured?	Yes	4	67 %
		No	2	33 %
	Was individual-level intervention exposure measured objectively?	Yes	0	0 %
		No	4	100 %
Bias in measurement of outcomes	Was the outcome measure valid and reliable?	Yes	7	47 %
		No	8	53 %
	Were the outcomes measured over a period of more than one week at each time point?	Yes	3	37.5 %
		No	5	62.5 %
	Were there multiple follow-up time points?	Yes	4	27 %
		No	11	73 %
Bias in selection of the reported result	Was a study protocol published?	Yes	0	0 %
		No	15	100 %
	Did the authors provide a clear and compelling justification for not publishing a study protocol?	Yes	0	0 %
		No	15	100 %

^a^This percentage is based on the total number of outcomes eligible for that particular signalling question, rather than the total number of outcomes included in this review

^b^These studies performed an appropriate analysis to control for differences between intervention and control groups at baseline

#### Domain 1: bias due to confounding

All outcomes either had a serious (*n* = 14) or critical risk of bias (*n* = 1) in this domain (see Table 4). According to MRC guidance [11], using multiple well-matched control groups strengthens the internal validity of natural experiments. Yet nine studies were judged as having poorly matched control sites and six studies did not use multiple control sites (see Table 5). Further, none of the outcomes had an appropriate analysis method that adjusted for all critically important confounding domains, thus increasing the risk of biased effect estimates in all outcomes (*n* = 15) (see Table 5).

Cohen et al. [38] had the lowest risk of bias in terms of control site matching. They attempted to match control parks to each intervention park using both built environment features and demographics of participants, provided a description of all matched variables for both control and intervention parks, and used multiple control parks. Moreover, matched variables were reasonably comparable across intervention and control parks. However, they failed to appropriately statistically adjust for a number of key confounding variables, resulting in serious risk of bias. These included differences in baseline outcome measurements and demographic characteristics for the systematic observation outcome, as well as a decline in observed organised physical activity activities and economic influences during follow-up.

#### Domain 2: bias in selection of participants into the study

The majority of outcomes in this domain either had a moderate (*n* = 7) or serious risk of bias (*n* = 7), whilst one outcome had a critical risk of bias (see Table 4). Reporting sufficient details about study participants is necessary to ascertain whether there are any differences between intervention and control groups that may confound findings [51], yet seven outcomes did not contain a clear and sufficient description of the sample (see Table 5). There was no reference to sample size calculations reported for any of the fifteen outcomes (see Table 5).

#### Domain 3: bias in measurement of interventions

Outcomes in this domain either had a low (*n* = 6), moderate (*n* = 4), or serious risk of bias (*n* = 5) (see Table 4). Whilst all studies described what was modified by the intervention (*k* = 12, *n* = 15), five studies did not sufficiently describe where it was implemented (*k* = 5, *n* = 6), and nine studies did not sufficiently describe how long it took to construct the intervention (*k* = 9, *n* = 11) (see Table 5). There was a risk of potential overlap between intervention construction and outcome measurements for three of the studies that did not sufficiently describe how long it took to construct the intervention (*k* = 3, *n* = 3) (see Table 5).

#### Domain 4: bias due to departures from intended interventions

The majority of outcomes had a low risk of bias in this domain (*n* = 8), whereas the remaining outcomes had a moderate (*n* = 3) or serious risk of bias (*n* = 4) (see Table 4). Out of the six self-report outcomes that did not sample directly from the intervention site, two did not measure intervention exposure (see Table 5). There is thus an increased risk in these two outcomes that changes in physical activity may not be attributable to changes in the built environment [52, 53]. All four outcomes that measured intervention exposure relied on self-report (see Table 5).

#### Domain 5: bias due to missing data

Most outcomes either had a low (*n* = 8) or moderate risk of bias (*n* = 4), whilst a minority had a serious risk of bias (*n* = 2). For one outcome, insufficient data were reported for response rates and missing participants to make a risk of bias judgement for this domain (see Table 4). Three out of seven self-report outcomes did not provide information on response rates. Overall response rates in the remaining self-report outcomes were as follows: 14 %, 31 %, 58 %, and 71 %.

#### Domain 6: bias in measurement of outcomes

One outcome that used systematic observation to measure physical activity had a low risk of bias in this domain (see Table 4). According to the ACROBAT-NRSI, this outcome is comparable to a well-performed randomised trial for this domain. The remaining outcomes either had a serious (*n* = 11) or critical risk of bias (*n* = 3) (see Table 4).

There was no evidence provided that any of the self-report outcome measures were valid and reliable (*n* = 7) (see Table 5). Three outcomes did not have any follow-up measurements conducted a sufficient duration after completion of the intervention to reduce the ‘novelty effect’ so that ‘normal’ physical activity levels were captured [19]. Conducting only one follow-up ‘may not provide a valid measure of change’ ([20]: p. 373), yet only four outcomes had multiple follow-up measurements (see Table 5). Out of the nine outcomes that used systematic observation, the majority of outcomes conducted observation periods at multiple times during the day, across multiple days on both weekdays and weekends (*n* = 8). However, five outcomes only observed physical activity over a period of one week or less at each time point, which is likely to increase the risk of invalid measurements due to variation in physical activity across different days and times of the week [54] (see Table 5).

#### Domain 7: bias in selection of the reported result

There was no reported study protocol and no clear and compelling justification for not publishing a study protocol in any of the included studies, which is why all outcomes across the twelve studies had a serious risk of bias in this domain (see Table 4). That is, there was no evidence of formulating precise data analysis plans before data were collected, thereby allowing post-hoc data analysis plans to capitalise on chance findings.

## Discussion

### Key findings

All outcomes in the best available natural experiments that have investigated the causal effect of changes to the built environment on physical activity had either an overall critical (*n* = 12) or serious (*n* = 3) risk of bias. Thus, according to principles of the ACROBAT-NRSI, four fifths of included outcomes are ‘too problematic to provide any useful evidence on the effects of intervention’ and one fifth ‘have some important problems’ ([21]: p. 12). Domains with the highest risk of bias across all outcomes were due to: confounding, measurement of outcomes, and selection of the reported result. Risk of bias was lower in other domains, but was still common.

### How this review compares to the current literature

Several reviews have concluded that there is sufficient evidence to show that modifying the built environment causes changes in physical activity levels [18, 22–25]. The present review is the first attempt at conducting a formal and thorough quantitative appraisal that focuses on the risk of bias in natural experiments in this area.

To the authors’ knowledge, Hunter et al. [19] is the only existing review to include a risk of bias appraisal of natural experiments in this area. They used a risk of bias tool designed for randomised trials, which is reflected in the finding that six out of twelve included studies had an unclear risk of bias. Nevertheless, they found that the remaining six studies had a high risk of bias, which is in line with the findings from our review. Their risk of bias assessment was more superficial as this was not the primary aim of their review.

Despite the high risk of bias in studies in this area, researchers have often prioritised other research directions. In a recent review of reviews in this area [15], the most common recommendation for future research was to examine potential moderators of the built environment-physical activity relationship. Whilst it is important to develop explanatory theoretical models of how the built environment influences physical activity behaviour, strengthening causal inferences has apparently received less focus to date. Exploring causal mechanisms was beyond the scope of the present review, particularly as many of the variables that are most strongly associated with physical activity levels (e.g., street connectivity, population density, land use [9]) were not targeted by interventions included in this review.

### Utility of the ACROBAT-NSRI for assessing natural experiments

This review only included natural experiments, as this research design is considered the most robust and feasible study design for strengthening causal inferences when evaluating population-level environmental interventions [11]. Given this, the credibility of our findings depends on the validity of the criteria for assessing the risk of bias in the ACROBAT-NRSI, the signalling questions used, and the studies selected for inclusion.

The ACROBAT-NRSI provides the most comprehensive coverage of bias for non-randomised studies [32]. However, it could be argued that the original ACROBAT-NRSI takes a fairly narrow perspective on causal inference by placing emphasis on RCTs as the “gold standard”, potentially overlooking the reality of the natural experimental context. For instance, the ACROBAT-NRSI favours objective outcome measures (e.g., systematic observation) over subjective outcomes (e.g., self-report). Yet an emphasis on the value of objective outcomes may disregard other complex or less quantifiable outcomes that still have potential to improve public health [55].

All new signalling questions were based on leading guidelines, primarily using the MRC guidance for natural experiments [11] and relevant literature in this area (see Additional file 1). Therefore, the extensions to the Cochrane tool are likely to be valid as they are based on methodological features of natural experiments that are known to increase the risk of bias. The validity of extensions to the ACROBAT-NRSI can be shown by examining a domain that had a high risk of bias due to new signalling questions added to the ACROBAT-NRSI: bias in selection of the reported result. All outcomes had a serious risk of bias in this domain because none of the included studies published a study protocol with a priori analyses specified. This standard is considered by MRC guidance [11] as important to minimise the risk of selective reporting bias and so its absence represents reasonable justification that all outcomes have a serious risk of bias in this domain [21].

In keeping with the original ACROBAT-NRSI, the seven bias domains were weighted equally. An alternative approach would have been to weight bias domains based on their relative importance for the outcome, and for influencing practical decisions in this field. However, what we have done is in line with the Cochrane approach. Throughout, we have aimed to follow the most robust procedure possible that is most defensible in terms of our ratings being objective and reproducible. Given that there has been little consistency in previous risk of bias tools that have weighted bias or quality domains [56], adjusting the principles of an established risk of bias tool by creating weighted bias domains would have been difficult to justify. If a weighting system were used, bias due to confounding would receive the highest weight because of lack of randomisation in natural experiments that increases the risk of confounding [22], as well as the problems associated with identifying adequate control groups. Our discussion of recommendations for future research reflects this by prioritising key issues in relation to poor control of confounding variables and inadequate control sites.

The ACROBAT-NRSI includes an optional component to judge the direction of the bias for each domain and overall risk of bias. Whilst in principle it would have been more informative to provide an additional analysis of the direction of bias, in practice it would have been difficult to reliably judge this. For example, although non-differential measurement error is likely to result in underestimates of intervention effects, it is also commonly found that poor measures contain systematic measurement error. It is difficult to ascertain whether such measures are likely to bias the estimated effect upwards or downwards. As we have aimed to follow the most robust procedure possible, we have therefore avoided judging this optional component due to difficulties in achieving high consensus. In line with this, previous reviews that have used the ACROBAT-NRSI have similarly not reported judgements for the direction of bias, suggesting they did not carry out these judgements or could not achieve reliable coding. This includes one systematic review in this area that looked at the effect of the urban environment on health in children and young people [57], and numerous other reviews within the field of public health [58–61]. Further, it is unlikely that analysing the direction of bias would have significantly altered the results of this review since there are similar numbers of problems detected that would affect the findings in an overall positive or negative direction. For instance, although selective reporting bias is likely to inflate positive findings, by contrast insufficient sample sizes are more likely to produce negative findings.

In sum, a number of key decisions were made that involved at least some degree of subjectivity when adapting the ACROBAT-NRSI for the present review. It is acknowledged that other approaches could have been taken that would be equally reasonable. However, it is highly likely that other reasonable approaches would have also identified key methodological flaws in the current evidence base according to leading guidance for conducting natural experimental studies in this area. Nonetheless, the present review has produced a comprehensive adaptation of an established risk of bias tool that can be used to assess risk of bias in future natural experiments in this field.

### Strengths and limitations

One potential limitation is the extent of subjectivity associated with coding individual signalling questions, and combining these questions to make a risk of bias judgement. The inter-rater agreement between two assessors for 10 outcomes was good (κ = 0.63), suggesting that the assessments reflected the features of studies, rather than features of those ratings. The specific signalling questions and guidance for their use, and the resulting risk of bias estimates are presented in Additional files 3 and 4 to provide transparency in the judgements made.

It is also possible that included studies may be somewhat inferior compared to studies that would have been obtained using an up-to-date systematic search of the literature. We believe that our approach is strong, for three reasons. First, all systematic reviews provided greater coverage than would a single systematic review as each review had different aims: one focused on the built environment and physical activity, one focused on the built environment in urban green spaces on physical activity, and one focused on policy and built environment effects on obesity-related outcomes. Thus, our approach yielded a range of different built environment intervention sites, including parks, urban greenways/trails, bicycle lanes, paths, vacant lots, and a senior citizen’s centre, therefore providing a complementary breadth of coverage of research in this area. These three reviews also used different databases to search for studies: only Medline was searched by all three reviews, two other databases were searched by two reviews, whilst six databases, which included coverage of urban studies, psychology and nursing literatures, were searched by one review only. As a result, all systematic reviews covered different literatures, as evidenced by the small degree of overlap in studies included in the three systematic reviews: seven of the twelve studies were included in only one of the reviews, four studies were included in two reviews, and only one study was included in all three reviews.

Second, there are already at least 31 physical activity-built environment systematic reviews [15], and the present approach allowed more effort to be devoted to a thorough consideration of risk of bias, rather than adding another systematic literature search to the large number previously conducted. Third, although it is possible that some more recent studies may exhibit lower risk of bias than those included in the present review, this does not explain why previous reviews recommended built environment interventions to increase physical activity on the basis of studies included in our review [23, 24].

### Implications for policy and practice

Many policy makers are beginning to advocate changing the built environment as an intervention to increase physical activity in the population [62]. Considering the high risk of bias identified in all studies included in the present review, it may be illuminating to compare the study appraisals between the present review and those that underpin policy guidelines. The NICE [29] guidelines in the UK are an example of one of the many policy guidelines that have recommended modifying the built environment to increase physical activity levels. These guidelines have been held in high regard across various health and non-health sectors [62], and have explicitly influenced other national policies in this area [27].

Although the NICE [29] guidelines were published before the majority of the included studies in this review, one study conducted by Merom et al. [44] informed these guidelines and was included in our review. Crucially, our review concluded that the primary physical activity outcome from this study had the highest overall risk of bias, as it had a critical risk of bias in two domains and serious risk of bias in three domains. In contrast, the NICE [29] guidelines judged that this study had an overall low risk of bias.

This disagreement can be explained by the appraisal tools used to evaluate the studies: the NICE [29] guidelines used the Graphical Appraisal Tool for Epidemiological studies (GATE) [63] that was revised and tailored to make it more relevant for public health interventions. However, there are a number of key issues that are missing from this tool, such as the use of a poorly matched single control site, the primary outcome being measured at one time-point four months after the intervention (thereby not controlling for seasonality), and no published study protocol. As a result, important limitations present in the Merom et al. [44] study were not detected due to their absence from the GATE tool used by NICE.

The results from the present review, using the most appropriate risk of bias tool, indicate that there is a lack of rigorous evidence that underpins policy recommendations in this field, such as those by NICE [29], in line with previous observations [62]. However, the present review focused more on internal validity and thus did not consider other factors, such as cost effectiveness, that need to be considered when developing policy guidance in public health. Although NICE [29] did recognise a number of methodological issues in the current evidence base, the aim of policy guidance is to make constructive recommendations for action now using the best available evidence. It would therefore be unrealistic, and potentially harmful, for policy makers to postpone recommendations and action for changing the built environment until more rigorous natural experiments are available, particularly as improving physical activity levels is unlikely to be a primary objective for urban planners. Rather, the findings from the present review highlight the need for researchers to conduct better natural experiments to inform the growing policy response in this area. This is even more important when considering the substantial cost of built environment interventions, which cost up to $5.5 million in the studies included in our review.

### Implications for research

Opportunities to conduct natural experiments in this area can be rare [62] so future research needs to acknowledge and improve the methodological flaws that have caused bias in research to date.

To initiate improvements, the following eight research priorities were identified from the present review (and in line with previous recommendations [19]) as design aspects of studies that need improvement:

1. Better matching of control sites and more nuanced use of graded exposure;
2. Use of multiple control sites;
3. Controlling for confounding domains;
4. Publishing study protocols with a priori analyses specified;
5. Use of adequate outcome measurements;
6. Better reporting of samples and interventions;
7. Sample size calculations; and;
8. Measuring exposure to the intervention at the individual level.

The domain with the second highest risk of bias for all included outcomes was ‘bias due to confounding’. This is most concerning for non-randomised studies since they are more susceptible to confounding than RCTs [22]. The first three research priorities identified by the present review (‘Better matching of control sites and more nuanced use of graded exposure’, ‘Use of multiple control sites’ and ‘Controlling for confounding domains’) are aimed at improving this bias domain.

When using a parallel-group design with ‘exposed’ and ‘unexposed’ comparison groups, future research must attempt to match the control and intervention sites to increase the likelihood that participants are comparable at baseline [11]. As well as matching on population demographics, future research should attempt to match control sites using objective measures of the built environment that have been found to correlate with physical activity levels, such as land use, population density, street connectivity, and physical infrastructure [9, 10].

MRC guidance suggests that graded measures of exposure, such as distance from the intervention, can provide appropriate comparison groups in natural experiments [11]. Four studies included in this review used graded measures of exposure. Whilst three of these studies reported a justification for why their chosen distances for classifying intervention and control groups was a reasonably valid measure of intervention exposure, all four studies used area-based spatial units. That is, they used comparison sites based on distance from the intervention, with intervention groups defined as those participants living in an area nearer to the intervention site. Future research should aim to develop more specific distance-based intervention and comparison groups that take into account differences in exposure between individuals who reside within the same geographical area (see Humphreys et al. [64] for further discussion).

Considering the difficulties associated with identifying suitable comparison groups, it is less likely that a single control site is sufficient to reduce confounding from key demographic and environmental variables. Using multiple control sites (including different types of control sites e.g., graded exposure, pre-intervention condition, matched control, synthetic control) offsets the variation in confounding variables across control sites and thus increases the likelihood of finding well balanced comparison groups [65]. Despite this, only half of included studies used multiple control sites. However, control sites should not be chosen adjacent to the intervention (e.g., [45]) to reduce the risk of contamination.

The difficulties in matching control and intervention groups both on observable and unobservable prognostic factors [11], as well as the absence of randomisation in natural experiments, means that baseline characteristics are likely to systematically differ across intervention groups. This is why future research should statistically test for baseline differences between intervention and control groups, particularly differences in age and gender as these characteristics are consistently correlated with physical activity [35]. They are also feasible to measure, even when directly observing physical activity behaviour [49, 66]. Appropriate statistical methods should be used to control for key confounding variables, as recommended by MRC guidance [11], such as propensity score weighting which was used by Cohen et al. [38, 40] for their self-report outcomes.

All studies had a serious risk of bias in relation to selection of the reported results because none of the studies published a pre-registered study protocol. Publishing a study protocol increases transparency and reduces the risk of selective reporting [67]. It also encourages researchers to address unforeseen issues [68], which is particularly important for natural experiments due to the lack of control that researchers have over the intervention. The high risk of bias in this domain can easily be resolved by publishing a pre-registered protocol describing the design, procedures and analysis that will be used in the study. Initiatives such as the Open Science Framework now allow this pre-registration at low cost or no cost [69].

The domain with the highest risk of bias for all included outcomes was ‘bias in measurement of outcomes’. The reason the majority of outcomes were at high risk of bias in this domain is likely attributable to the effort and cost associated with available methodology for measuring and obtaining repeated measurements of physical activity. Also noteworthy, some studies that relied on self-report had low overall response rates (14 % and 31 %), which is particularly common in population studies of physical activity [10]. Triangulation between observational measures and self-report or accelerometer data provides reassurance that findings are robust to the different types of bias associated with each individual method of measurement. Whilst using systematic observation is generally considered a process measure that assesses usage of the built environment rather than changes in physical activity behaviour per se, observations provide advantages of objectivity, flexibility and low participant burden [70]. They also remove issues of response rates and subjectivity associated with self-report [70], accuracy concerns when using accelerometers [71], and possible reactivity of measurement [72]. However, the common problems associated with systematic observation can only be improved once less costly and less labour-intensive methodology is developed, possibly based on photography or video technology for observing physical activity [73].

Poor reporting was a common issue for many studies in the present review, particularly descriptions of samples and interventions. Poor reporting was generally penalised in the present review since this can be an indicator of the risk of bias in that study [74]. Clear and complete reporting of a study is necessary to diminish any ambiguities in the study’s methodology and therefore assess validity of the findings [75]. Future research may find it useful to follow established guidelines such as STROBE checklist for non-randomised studies [76] until more specific guidelines are developed for this research area.

None of the included studies made reference to sample size calculations. Without an appropriate sample size calculation, studies are at an increased risk of type II errors due to an inappropriately small sample size to detect an effect. Alternatively, studies may have larger numbers of observations than is required to adequately power a study, resulting in overly expensive studies, or possibly having too few comparison sites due to limited resources being spent on unnecessary observations being made at those sites. Sample size calculations are particularly difficult when using systematic observation as there is limited information regarding typical physical activity behaviour in different built environment spaces, on different days and times of the week. One way of performing sample size calculations would be to carry out visits to the target area before the study period to estimate the duration of observation periods at different time of day/day of week necessary to provide narrow confidence intervals for that specific area [70]. Whilst none of the studies included in the present review appeared to have conducted sample size calculations, more recent natural experiments provide examples of methods for calculating the appropriate sample size (e.g., [77, 78]).

It is important to measure intervention exposure accurately as it enables us to spatially match changes in physical activity with actual exposure to the built environment intervention. Yet none of the self-report outcomes that were conducted away from the intervention site measured intervention exposure objectively. Relying on self-report to measure individual-level intervention exposure increases the risk of invalid estimations [36]. Objective measurements, such as global positioning system (GPS) monitors, could therefore be used to quantify the extent to which changes in physical activity (at least in a sub-sample of participants) are specifically attributable to exposure to the built environment intervention of interest.

Suggestions have been made for the issues identified in the present review, in relation to: (a) what can feasibly be improved at this stage in the research area, and (b) what requires further investigation before improvements are possible (see Additional file 6).

Although external validity was not the focus of the present review, it is worth highlighting that most of the included studies were conducted in the US, which is common in research on the built environment and physical activity [15]. This is an issue because there are numerous factors that often vary between different countries that can affect findings. For example, there are huge variations in climate across different parts of the world that influence physical activity levels [79]. Many cities in Europe also have higher population density and more mixed land use than is typical of cities in the US [80], many of which were more influenced by car usage. Other examples of potential confounders include higher obesity rates in the US compared to Europe [81], as well as differences in physical activity patterns [80]. Thus, whist natural experiments offer the advantage of high levels of external validity for the setting and population that is affected, more research outside of the US is needed so that findings may be generalised to other countries.

## Conclusion

Researchers are now recognising the importance of conducting natural experiments to strengthen causal inferences when evaluating population-level interventions [11, 13]. We argue that methodologically stronger future study is required to underpin policy and practitioner recommendations. Eight research priorities were identified to help reduce the risk of bias in future natural experiments and which reflect the reality of the natural experimental context: (1) better matching of control sites and more nuanced use of graded exposure; (2) use of multiple control sites; (3) controlling for confounding domains; (4) publishing study protocols with a priori analyses specified; (5) use of adequate outcome measurements; (6) better reporting of samples and interventions; (7) sample size calculations; and (8) measuring exposure to the intervention at the individual-level. Whilst some of these issues are attributable to the available methodology and general difficulties of conducting rigorous natural experiments [11], clear and pragmatic suggestions have been proposed to improve studies in this area.

Researchers and policy makers alike have gradually shown increased support to implement expensive built environment interventions to improve population-level physical activity levels [61]. This growing interest increases the need to better test the hypothesis that built environment interventions are effective in increasing physical activity levels. The present review suggests that existing studies are methodologically flawed in a number of key bias domains. This review highlights suggested areas for improving methodological rigour that need to be taken into account in the next generation of natural experiments.

## Additional files

Additional file 1: Description of guidance and evidence that all new signalling questions added to the ACROBAT-NRSI were based on. (DOCX 28 kb)
Additional file 2: Overview of signalling questions added to the ACROBAT-NRSI. (DOCX 26 kb)
Additional file 3: Accompanying guidance document used to help assessors judge the risk of bias in each bias domain and overall risk of bias. (DOCX 29 kb)
Additional file 4: The adapted ACROBAT-NRSI used for the present review. (DOCX 37 kb)
Additional file 5: Detailed summary of the key characteristics and results of included studies. (DOCX 29 kb)
Additional file 6: Summary of suggestions to improve future research in this area. (DOCX 26 kb)

## Abbreviations

ACROBAT-NRSI: A cochrane risk of bias assessment tool: for non- randomized studies of interventions
MRC: Medical research council
PRISMA: Preferred reporting items for systematic reviews and meta-analyses
RCT: Randomised controlled trial
SOPARC: System for observing play and recreation in communities
SOPLAY: System for observing play and leisure activity in youth
UK: United Kingdom
US: United States
