# Supplementary material for: The effect of changing the built environment on physical activity: a quantitative review of the risk of bias in natural experiments
Source: Int J Behav Nutr Phys Act. 2016 Oct 7;13:107. doi: 10.1186/s12966-016-0433-3 (PMC5055702; doi:10.1186/s12966-016-0433-3)
Supplement: Additional file 1: — Description of guidance and evidence that all new signalling questions added to the ACROBAT-NRSI were based on. (DOCX 28 kb) [file 12966_2016_433_MOESM1_ESM.docx]

**Additional file 1. Description of guidance and evidence that all new signalling questions added to the ACROBAT-NRSI were based on ^[[1]](#footnote-1)^**

***Domain 1: Bias due to confounding***

Confounding occurs when one or more variables also explain the observed relationship between exposure and outcome. As recommended by the ACROBAT-NRSI [22], a list of the critically important confounding domains in this research area was identified using scoping reviews of the literature, including the following four confounding domains:

1. *Differences in baseline outcome measurements.* This is because dissimilar measurements between intervention groups increases the risk that participants may not be comparable for the outcome since participants are not randomly allocated in natural experiments [14] (1.5);
2. *Differences in baseline demographic characteristics.* Particularly age and gender as these characteristics are consistently correlated with physical activity [35], and are also feasible to measure even when systematically observing physical activity behaviour [49,66] (1.6);
3. *Any unusual events.* This includes any cultural or religious events, sporting events and music festivals (1.7); and,
4. *Socioeconomic or political influences.* E.g., crime and conflict that could influence physical activity behaviour (1.8).

Weather conditions were added as an additional confounding for systematic observation outcomes or live data collected within a specified period of time, such as accelerometers [82]. Precipitation and temperature should be considered as rain and cold weather have consistently been acknowledged as barriers to physical activity [79] (1.13, 1.14). Although this list of confounding domains is by no means exhaustive, they represent the main confounding domains most relevant to studies in this area.

Control or comparison groups that are unexposed to the intervention are crucial in strengthening the internal validity of natural experiments and reducing the risk of bias due to confounding, because they provide a counterfactual of what physical activity levels would have been without the intervention [11]. Control groups for studies in this research area tend to include sites that have not undergone any change to the built environment, but should be well matched to the intervention site in terms of size, features, amenities and population, whilst existing far enough apart to reduce contamination whereby participants are inadvertently exposed to the control site [19] (1.15).

Adequately matched control groups reduce the likelihood that any changes in the intervention site are influenced by other factors aside from the intervention, thus reducing the risk of confounding [11] (1.16, 1.19, 1.20). This should include variables based on features of the built environment that correlate with physical activity, such as land use, population density, street connectivity and physical infrastructure [9,10] (1.17). Researchers should also attempt to match intervention and control groups based on demographic characteristics, particularly differences in age and gender as these demographics are consistently correlated with physical activity [35] (1.18).

Considering the difficulties associated with identifying suitable comparison groups, it is less likely that a single control site is sufficient to reduce confounding from key demographic and environmental variables. Using multiple control sites (including different types of control sites e.g., pre-intervention condition, matched control, synthetic control) offsets the variation in confounding variables across control sites and thus increases the likelihood of finding comparable control groups [65] (1.21).

Given that control sites are not necessarily under the control of researchers, it is important that control sites are monitored to ensure that they do not undergo any significant changes during the study, as any changes could cause confounding unless appropriately adjusted for in the analysis (1.22, 1.23).

***Domain 2: Bias in selection of participants into the study***

This bias domain refers to the exclusion of eligible participants that biases the outcome [22]. Without an appropriate sample size calculation, studies are at an increased risk of type 1 errors due to an inappropriately small sample. Alternatively, studies may have larger numbers of observations than is required to adequately power a study, resulting in overly expensive studies, or possibly having too few comparison sites due to too many observations being made at those sites (2.1).

As recommended by MRC guidelines, providing explicit sampling criteria by clearly describing the participants, variables and outcomes of interest to the study is good practice for natural experiments [11] (2.2). Also, reporting sufficient details about study participants is recommended by the STROBE checklist for non-randomised studies [76]. A clear and sufficient description of the sample is necessary to determine whether there are any significant differences between intervention and control groups that may confound findings [51] (2.3).

The final adaptation in this bias domain was to split the signalling question “Was selection into the study unrelated to intervention or unrelated to outcome?”, because this relates to two distinct issues: first, whether participants self-selected into the intervention group (2.4), and second, whether there are any key differences relating to the outcome between intervention and control groups (2.5).

***Domain 3: Bias in measurement of interventions***

Bias in this domain occurs when intervention status is misclassified; that is, when errors in measuring participants exposure to the intervention biases the estimated effect of the intervention [22]. For some studies it may only be possible to sample from a proportion of the intervention site, so for these studies it is important that the sampling sites are selected using probability-based sampling to reduce systematic bias [20] (3.1). If probability-based sampling is not possible or appropriate, then the selection of the sampling site needs to be appropriately justified to capture a valid representation of the whole intervention (3.2).

Once intervention sites have been selected, a clear description of the intervention is necessary for the purposes of replication and clarity about what features of the built environment are under investigation. In line with the Template for Intervention Description and Replication (TIDieR) checklist and guide for improved reporting of interventions [83], the intervention should be clearly described in terms of what was modified (3.3), where the intervention was implemented (3.4) and how long it took to construct the intervention (3.5). Omitting a clear description of how long it took to construct the intervention poses an increased risk of bias if intervention construction could potentially overlap with outcome measurements (3.6).

Signalling questions 3.7, 3.8 and 3.9 were not applicable for systematic observation outcomes because intervention status (i.e. intervention or control group) is already defined by the location of the observations (i.e. intervention or control site).

***Domain 4: Bias due to departures from intended interventions***

This bias domain refers to systematic differences between intervention and control groups due to departures from the intended intervention [22]. Although the ACROBAT-NRSI recommends listing all possible co-interventions that could differ between intervention and control groups [22], this was not carried out since this list would be exhaustive. Instead, it was presumed that researchers would explicitly report any other known physical activity interventions that occurred during the study period. For example, Tester and Baker [46] evaluated a built environment intervention and also a recreation program improvement initiative, of which the latter would need to be accounted for (4.1, 4.2).

A particularly common issue associated with evaluating changes in the built environment is potential delays in implementation of the intervention [84]. Even though delays may not directly increase the risk of bias, it becomes an issue if it impacts upon the study design [20] (4.3). For example, delays that prevent or disrupt researchers from collecting post-intervention data before the researcher’s contract expires increases the risk of bias in the measurement of outcomes [85].

Even if the intervention is implemented as planned and in accordance with the pre-specified time frame, if participants were not truly exposed to the intervention then we cannot attribute increases in physical activity to changes in the built environment [53]. For example, when researchers use larger areas for geographic definitions of a site, variability in exposure to different locations within these areas is more likely to be missed [36]. Therefore, as recommended by McCormack and Shiell [10], individual-level outcomes that do not measure directly from the intervention site should attempt to measure individual-level intervention exposure to determine how much of the change in physical activity is attributable to participants’ exposure to the built environment of interest (4.5).

It is important to measure intervention exposure accurately as it enables us to spatially match changes in physical activity with actual exposure to the built environment intervention. Relying on self-report to measure individual-level intervention exposure increases the risk of invalid estimations [36]. Objective measurements, such as global positioning system (GPS) monitors, could therefore be used to quantify the extent to which changes in physical activity are specifically attributable to exposure to the built environment intervention of interest (4.6).

***Domain 5: Bias due to missing data***

Studies that have missing data increase the risk of selection bias, thus resulting in a misrepresented sample [22]. For instance, individuals who are more physically active may be more likely to return physical activity questionnaires than less physically active individuals, which would result in a misrepresentative sample. The ACROBAT-NRSI includes the signalling question ‘Are outcome data reasonably complete?’. Three further signalling questions were added to provide a detailed breakdown of response rates for those eligible at baseline (5.1a), at follow-up (5.1b), and the overall response rate (5.1c). For repeated cross-sectional outcomes, overall response rates were defined as the average of all response rates at each time point. For within-person longitudinal outcomes, overall response rates were defined as participants from the initial sample who completed surveys at all time points.

The ACROBAT-NRSI recommends that assessors should provide guidance for what is meant by ‘reasonably complete’ data. In terms of response rates, there is currently no clear threshold that has been consistently agreed upon in the literature. Nevertheless, a response rate of 70% was deemed a reasonable threshold in line with previous suggestions [86], although this decision is somewhat a matter of opinion and should not be considered a gold standard in this field.

***Domain 6: Bias in measurement of outcomes***

Bias can occur when there are errors in measuring outcomes of the intervention [22]. Firstly, it is important that outcomes are clearly operationalised (e.g., the times and days when observations were carried out) [34,51] (6.1). A clear description of outcome measures will help improve the consistency of outcome measurement to reduce the risk of misclassification bias [67]; in which errors result from inaccurate measurement of physical activity.

As recommended by MRC guidelines for natural experiments [11], outcome measures that are validated and reliable, ideally for the target population [51], are necessary to avoid using measures that are inaccurate and vary over time, thus reducing possible misclassification bias (6.2). Established measures that are validated and reliable, such as SOPARC methodology for systematic observation [49], should be used. If not using established methodology, then authors should provide details about the validity and reliability of their chosen outcome measures.

When using systematic observation, obtaining multiple baseline and post-intervention measurements is important to reduce random error [87] (6.4). Given the variation in physical activity levels across different days and times of the week [54], outcomes should be measured at multiple time points across the course of a day (6.5), across multiple days (6.6), on both weekdays and weekends (6.7), and for over a period of more than one week at each time point (6.8). These measurements should be conducted at the same time of day at baseline and follow-up to provide valid comparisons by reducing bias due to time and day variation [19] (6.9).

Physical activity levels often also vary across different seasons [88], so follow-up outcome measurements should be conducted at the same time of year as baseline measurements to minimise bias due to seasonal variation [19] (6.10). As well as obtaining multiple measurements within each time point, it is also important to obtain multiple follow-up measurements at different time points because built environment interventions are long-term, permanent projects and usage often fluctuates over time [19] (6.11). For this reason, conducting only one follow-up ‘may not provide a valid measure of change’ [20: p. 373].

It is also important to consider the length of time from completion of the intervention to the first follow-up time point. According to Hunter et al. (2015), there should be a sufficient duration of follow-up after completion of the intervention to reduce the ‘novelty effect’ so that ‘normal’ physical activity levels are captured. Hunter et al. [19] and Mayne et al. [20] proposed that a minimum of 12-month follow-up is required to obtain a valid assessment of physical activity behaviour change (6.12).

Finally, it is important that participants are unaware of being assessed to reduce the risk of performance biases, particularly social desirability bias where participants adjust their physical activity levels to be portray a positive image of themselves [89] (6.13). Performance bias can lead to contamination or confounding [90]. Although blinding participants would be difficult for some outcomes in this area, particularly physical activity self-report questionnaires and interviews, it is important that studies use blinding methods where possible to reduce the risk of performance bias (e.g., using covert observations).

***Domain 7: Bias in selection of the reported result***

This domain refers to the selective reporting of fully reported results [22]. A pre-registered study protocol specifying the objectives and methods of the study should be published, unless there is a clear and compelling justification for not doing so (7.1, 7.2). Publishing a study protocol increases transparency and reduces the risk of selective reporting [66]. It also encourages researchers to address unforeseen issues [60], which is particularly important for natural experiments due to the lack of control that researchers have over the intervention. Therefore, all of the study’s pre-specified analysis and outcomes should be conducted and reported in the pre-specified way (7.3), if not then a clear and compelling justification should be provided for not doing so (7.4).

***Overall risk of bias***

In the ACROBAT-NRSI, the authors stated the following:

‘… in practice some ‘Serious’ risks of bias (or ‘Moderate’ risks of bias) might be considered to be additive, so that ‘Serious’ risks of bias in multiple domains can lead to an overall judgement of ‘Critical’ risk of bias (and, similarly, ‘Moderate’ risks of bias in multiple domains can lead to an overall judgement of ‘Serious’ risk of bias)’ [21: p. 12].

Therefore, to maintain consistency throughout the analysis, if an outcome has a particular risk of bias (e.g. “serious”) in at least four domains, then this outcome has an overall risk of bias of the next highest level (e.g. “critical”). The threshold was judged at four because this was over 50% of the bias domains and it is thus reasonable to assume that the accumulation of bias in four domains would be sufficient to cause an overall higher risk of bias.

**Additional references**

82. Salmon J, Owen N, Crawford D, Bauman A, Sallis JF. Physical activity and sedentary behavior: a population-based study of barriers, enjoyment, and preference. Health Psychology. 2003;22(2):178-188.

83. Hoffmann TC, Glasziou PP, Boutron I, Milne R, Perera R, Moher D, Altman DG, Barbour V, Macdonald H, Johnston M, Lamb SE. Better reporting of interventions: template for intervention description and replication (TIDieR) checklist and guide. BMJ. 2014;348:1756-1833.

84. Ogilvie D, Giles-Corti B, Hooper P, Yang L, Bull F. Methods for researching the physical activity impacts of ‘natural experiments’ in modifying the built environment. Journal of Physical Activity & Health. 2010;7(Suppl):S341-3.

85. Ogilvie D, Cummins S, Petticrew M, White M, Jones A, Wheeler K. Assessing the evaluability of complex public health interventions: five questions for researchers, funders, and policymakers. Milbank Quarterly. 2011;89(2):206-25.

86. Mundy D. A question of response rate. Science editor. 2002;25(1):25-26.

87. Owen N, Humpel N, Leslie E, Bauman A, Sallis JF. Understanding environmental influences on walking: review and research agenda. American Journal of Preventive Medicine. 2004;27(1):67-76.

88. Plasqui G, Westerterp KR. Seasonal variation in total energy expenditure and physical activity in Dutch young adults. Obesity Research. 2004;12(4):688-94.

89. Krauth J. Experimental design: a handbook and dictionary for medical and behavioral research: Elsevier; 2000.

90. Mayer D. Essential evidence-based medicine: Cambridge University Press; 2004.

1. Numbers in brackets refer to the relevant signalling question(s) in the adapted ACROBAT-NRSI (Appendix III). [↑](#footnote-ref-1)
