# Supplementary material for: The effect of changing the built environment on physical activity: a quantitative review of the risk of bias in natural experiments
Source: Int J Behav Nutr Phys Act. 2016 Oct 7;13:107. doi: 10.1186/s12966-016-0433-3 (PMC5055702; doi:10.1186/s12966-016-0433-3)
Supplement: Additional file 2: — Overview of signalling questions added to the ACROBAT-NRSI. (DOCX 26 kb) [file 12966_2016_433_MOESM2_ESM.docx]

**Additional file 2. Overview of signalling questions added to the ACROBAT-NRSI**

| **Bias domain** | **New signalling question** |
| --- | --- |
| Bias due to confounding | Did the authors control for… |
|  | 1.5. … differences in baseline outcome measurements? |
|  | 1.6. … differences in baseline demographic characteristics? |
|  | 1.7. … any unusual events? (E.g., cultural or religious events, sporting events, music festivals etc.) |
|  | 1.8. … socioeconomic or political influences? (E.g., natural disasters, crime and conflict etc.) |
|  | Questions 1.13 and 1.14 are only applicable for outcomes using systematic observation or live data collected within a specified period of time (e.g., accelerometers) |
|  | 1.13. Were weather conditions similar across all observation periods? |
|  | 1.14. **If N or PN to 1.13**: Did the authors use an appropriate analysis method that adjusted for differences in weather conditions? |
|  | 1.15. Did the authors attempt to match the control site with the intervention site? |
|  | **If Y or PY to 1.15:** |
|  | 1.16. How did they match the control site with the intervention site? |
|  | 1.17. Were the intervention and control site matched using any variables based on features of the built environment? |
|  | 1.18. Were the intervention and control site matched using any variables based on demographics? |
|  | 1.19. Is the control site well matched to the intervention site? |
|  | **If N or PN to 1.20:** |
|  | 1.20. Did the authors use an appropriate analysis method that adjusted for all the critically important differences between control and intervention sites? |
|  | 1.21. Were there multiple control sites? |
|  | 1.22. Is it unlikely that the control site underwent any significant changes during the study period that did not similarly occur in the intervention site and could influence the outcome? |
|  | 1.23. **If N or PN to 1.22:** Did the authors use an appropriate analysis method that adjusted for these significant changes? |
| Bias in selection of participants into the study | 2.1. Is there a fully justified sample size calculation? |
|  | 2.2. Are the sampling criteria for participants clearly described? |
|  | 2.3. Is there a clear and sufficient description of the sample? |
|  | 2.4. Was selection into the study unrelated to intervention? |
|  | 2.5. Was selection into the study unrelated to outcome? |
| Bias in measurement of interventions | Questions 3.1 and 3.2 are only applicable to studies that **did not** sample from the whole intervention site |
|  | 3.1. Was the sampling site selected using probability-based sampling? |
|  | 3.2. **If N or PN to 3.1**: Was the selection of the sampling site appropriately justified to capture a valid representation of the whole intervention? |
|  | Did the authors describe… |
|  | 3.3. … what was modified in the intervention? |
|  | 3.4. … where the intervention was implemented? |
|  | 3.5. … how long it took to construct the intervention? |
|  | 3.6. **If N or PN to 3.5:** Is it unlikely that intervention construction could overlap with outcome measurements? |
| Bias due to departures from intended interventions | 4.3. Is it unlikely that any delays or changes in intervention construction impacted upon the study? |
|  | 4.4. **If N or PN to 4.3**: Were adjustment techniques used that are likely to correct for these issues? |
|  | Questions 4.5 and 4.6 are **not** applicable for population-level outcomes or any outcomes measured directly from the intervention site (e.g., intercept surveys) |
|  | 4.5. Was individual-level intervention exposure measured? |
|  | 4.6. **If Y or PY to 4.7**: Was individual-level intervention exposure measured objectively? |
| Bias due to missing data | 5.1a. What was the initial response rate of participants eligible at baseline? |
|  | 5.1b. What was the response rate of participants sampled at follow-up? |
|  | 5.1c. What was the overall response rate? |
| Bias in measurement of outcomes | 6.1. Was the outcome clearly and sufficiently described? |
|  | 6.2. Was the outcome measure valid and reliable? |
|  | Questions 6.4 to 6.9 are only applicable for outcomes using systematic observation or live data collected within a specified period of time (e.g., accelerometers) |
|  | 6.4. Were there multiple baseline and follow-up observation periods? |
|  | **If Y or PY to 6.4:** |
|  | 6.5. Were the outcomes measured at multiple times across the course of a day? |
|  | 6.6. Were the outcomes measured across multiple days? |
|  | 6.7. Were the outcomes measured on both weekdays and weekends? |
|  | 6.8. Were the outcomes measured over a period of more than one week at each time point? |
|  | 6.9. Were follow-up outcome measurements conducted at the same time of day as baseline outcome measurements? |
|  | 6.10. Were any follow-up outcome measurements conducted at the same time of year as baseline outcome measurements? |
|  | 6.11. Were there multiple follow-up time points? |
|  | 6.12. Were any follow-up outcome measurements conducted sufficiently after completion of the intervention to reduce the ‘novelty effect’? |
|  | 6.13. Were participants unaware of being assessed for the purposes of the study? |
| Bias in selection of the reported result | 7.1. Was a study protocol published? |
|  | 7.2. **If N or PN to 7.1**: Did the authors provide a clear and compelling justification for not publishing a study protocol? |
|  | 7.3. **If Y or PY to 7.1**: Are all of the study’s pre-specified analysis and outcomes conducted and reported in the pre-specified way? |
|  | 7.4 **If N or PN to 7.3**: Did the authors provide a clear and compelling justification for not conducting and reporting the study’s pre-specified analysis and outcomes in the pre-specified way? |
| Y = Yes; PY = Probably yes; N = No; PN = Probably no | |
