# Supplementary material for: The effect of changing the built environment on physical activity: a quantitative review of the risk of bias in natural experiments
Source: Int J Behav Nutr Phys Act. 2016 Oct 7;13:107. doi: 10.1186/s12966-016-0433-3 (PMC5055702; doi:10.1186/s12966-016-0433-3)
Supplement: Additional file 3: — Accompanying guidance document used to help assessors judge the risk of bias in each bias domain and overall risk of bias. (DOCX 29 kb) [file 12966_2016_433_MOESM3_ESM.docx]

**Additional file 3. Accompanying guidance document used to help assessors judge the risk of bias in each bias domain and overall risk of bias**

**1. Bias due to confounding**

| Low risk of bias (the study is comparable to a well-performed randomized trial with regard to this domain) | (Unusual) No confounding is expected. |
| --- | --- |
| Moderate risk of bias (the study is sound for a non-randomized study with regard to this domain but cannot be considered comparable to a well- performed randomized trial) | Confounding expected, all known critically important confounding domains appropriately measured and adjusted for;  and  Reliability and validity of measurement of all critically important domains were sufficient that we do not expect serious residual confounding. |
| Serious risk of bias (the study has some important problems) | At least one known critically important domain not appropriately measured, or not adjusted for;  or  Reliability or validity of measurement of a critically important domain was low enough that we expect serious residual confounding. |
| Critical risk of bias (the study is too problematic to provide any useful evidence on the effects of intervention) | Confounding inherently not controllable, or use of negative controls strongly suggests unmeasured confounding. |
| No information on which to base a judgement about risk of bias for this domain | No information on whether confounding might be present. |

Any of the following means that the study is **at least at serious risk of bias**:

- There was a single control site that was not well matched to the intervention site;

**and**

- - The authors did not use an appropriate analysis method that adjusted for all the critically important differences between control and intervention sites

or

- There were multiple control sites;

**and**

- - None of these sites were well matched to the intervention site;

**and**

- - The authors did not use an appropriate analysis method that adjusted for all the critically important differences between control and intervention sites

Any of the following means that the study is at **critical risk of bias**:

- The control site underwent significant changes during the study period that did not similarly occur in the intervention site and could influence the outcome;

**and**

- - The authors did not use an appropriate analysis method that adjusted for these significant changes

**The following only apply to systematic observation outcomes or live data collected within a specified period of time (e.g., accelerometers):**

Any of the following means that the study is at **critical risk of bias**:

- Weather conditions were not similar across all observation periods and this was not appropriately adjusted for

**2. Bias in selection of participants into the study**

| Low risk of bias (the study is comparable to a well-performed randomized trial with regard to this domain) | All participants who would have been eligible for the target trial were included in the study and start of follow up and start of intervention coincide for all subjects. |
| --- | --- |
| Moderate risk of bias (the study is sound for a non-randomized study with regard to this domain but cannot be considered comparable to a well- performed randomized trial) | Selection into the study may have been related to intervention and outcome, but the authors used appropriate methods to adjust for the selection bias;  or  Start of follow up and start of intervention do not coincide for all participants, but (a) the proportion of participants for which this was the case was too low to induce important bias; (b) the authors used appropriate methods to adjust for the selection bias; or (c) the assessors are confident that the rate (hazard) ratio for the effect of intervention remains constant over time. |
| Serious risk of bias (the study has some important problems) | Selection into the study was related to intervention and outcome;  or  Start of follow up and start of intervention do not coincide, and a potentially **important amount** of follow-up time is missing from analyses, and the rate ratio is not constant over time.  *N.B. For studies in this area, it is generally agreed in the literature that a follow-up period of at least 12 months from construction of the intervention is needed to capture “normal activity”. Therefore, assessors need to be lenient on the amount of follow-up time missing. If the* ***first*** *follow-up time point is significantly longer than 12 months, then this study can be judged at least at serious risk of bias for this domain.* |
| Critical risk of bias (the study is too problematic to provide any useful evidence on the effects of intervention) | Selection into the study was strongly related to intervention and outcome;  or  A substantial amount of follow-up time is likely to be missing from analyses, and the rate ratio is not constant over time. |
| No information on which to base a judgement about risk of bias for this domain | No information is reported about selection of participants into the study or whether start of follow up and start of intervention coincide. |

Any of the following means that the study is **at least at moderate risk of bias**:

- There was no attempt at a sample size calculation;

or

- The sampling criteria for participants were not clearly described

Any of the following means that the study is **at least at serious risk of bias**:

- There was not a clear and sufficient description of the sample

**3. Bias in measurement of interventions**

| Low risk of bias (the study is comparable to a well-performed randomized trial with regard to this domain) | Intervention status is well defined and based solely on information collected at the time of intervention. |
| --- | --- |
| Moderate risk of bias (the study is sound for a non-randomized study with regard to this domain but cannot be considered comparable to a well- performed randomized trial) | Intervention status is well defined but some aspects of the assignments of intervention status were determined retrospectively. |
| Serious risk of bias (the study has some important problems) | Intervention status is not well defined, or major aspects of the assignments of intervention status were determined in a way that could have been affected by knowledge of the outcome. |
| Critical risk of bias (the study is too problematic to provide any useful evidence on the effects of intervention) | (Unusual) An extremely high amount of misclassification of intervention status, e.g. because of unusually strong recall biases. |
| No information on which to base a judgement about risk of bias for this domain | No definition of intervention or no explanation of the source of information about intervention status. |

Any of the following means that the study is **at least at moderate risk of bias**:

- The authors did not describe what was modified in the intervention;

or

- The authors did not describe where the intervention was implemented;

or

- The authors did not describe how long it took to construct the intervention;

**and**

- - There is a risk that intervention construction could overlap with outcome measurements

Any of the following means that the study is **at least at serious risk of bias**:

- The authors did not describe **two or more** of the following:
- The authors did not describe what was modified in the intervention;

or

- The authors did not describe where the intervention was implemented;

or

- The authors did not describe how long it took to construct the intervention;

**and**

- - There is a risk that intervention construction could overlap with outcome measurements;

or

- The intervention site was not selected using probability-based sampling and the selection of the sampling site was **not** appropriately justified to capture a valid representation of the whole intervention

Any of the following means that the study is **at least at critical risk of bias**:

- The authors did not describe what was modified in the intervention;

**and**

- The authors did not describe where the intervention was implemented;

**and**

- The authors did not describe how long it took to construct the intervention;

**and**

- - There is a risk that intervention construction could overlap with outcome measurements

**4. Bias due to departures from intended interventions**

| Low risk of bias (the study is comparable to a well-performed randomized trial with regard to this domain) | No bias due to departure from the intended intervention is expected, for example if both the intervention and comparator are implemented over a short time period, and subsequent interventions are part of routine medical care, or if the specified comparison relates to initiation of intervention regardless of whether it is continued. |
| --- | --- |
| Moderate risk of bias (the study is sound for a non-randomized study with regard to this domain but cannot be considered comparable to a well- performed randomized trial) | Bias due to departure from the intended intervention is expected, and switches and co-interventions are appropriately measured and adjusted for in the analyses. Alternatively, most (but not all) departures from intended intervention reflect the natural course of events after initiation of intervention. |
| Serious risk of bias (the study has some important problems) | Switches in treatment or co-interventions are apparent and are not adjusted for in the analyses. |
| Critical risk of bias (the study is too problematic to provide any useful evidence on the effects of intervention) | Substantial departures from the intended intervention are present and are not adjusted for in the analysis. |
| No information on which to base a judgement about risk of bias for this domain | No information is reported on whether there is departure from the intended intervention. |

Any of the following means that the study is **at least at serious risk of bias**:

- Delays in intervention construction impacted upon the study design;

**The following only apply to self-report outcomes conducted away from the intervention site:**

Any of the following means that the study is **at least at moderate risk of bias**:

- Individual-level intervention exposure was measured but was **not** measured objectively

Any of the following means that the study is **at least at serious risk of bias**:

- Delays in intervention construction impacted upon the study design;

or

- Individual-level intervention exposure was not measured

**5. Bias due to missing data**

| Low risk of bias (the study is comparable to a well-performed randomized trial with regard to this domain) | Data were reasonably complete;  or  Proportions and reasons of missing participants were similar across intervention groups;  or  Analyses that addressed missing data are likely to have removed any risk of bias. |
| --- | --- |
| Moderate risk of bias (the study is sound for a non-randomized study with regard to this domain but cannot be considered comparable to a well- performed randomized trial) | Proportions of missing participants differ across interventions;  or  Reasons for missingness differ minimally across interventions;  or  There is no information on the proportions of missing participants and reasons for missingness across interventions;  and  Missing data were not addressed in the analysis. |
| Serious risk of bias (the study has some important problems) | Proportions of missing participants differ substantially across interventions;  or  Reasons for missingness differ substantially across interventions;  and  Missing data were addressed inappropriately in the analysis;  or  The nature of the missing data means that the risk of bias cannot be removed through appropriate analysis. |
| Critical risk of bias (the study is too problematic to provide any useful evidence on the effects of intervention) | (Unusual) There were critical differences between interventions in participants with missing data that were not, or could not, be addressed through appropriate analysis. |
| No information on which to base a judgement about risk of bias for this domain | No information is reported about missing data or the potential for data to be missing. |

**6. Bias in measurement of outcomes**

| Low risk of bias (the study is comparable to a well-performed randomized trial with regard to this domain) | The methods of outcome assessment were comparable across intervention groups;  and  The outcome measure was unlikely to be influenced by knowledge of the intervention received by study participants (i.e. is objective) or the outcome assessors were unaware of the intervention received by study participants;  and  Any error in measuring the outcome is unrelated to intervention status. |
| --- | --- |
| Moderate risk of bias (the study is sound for a non-randomized study with regard to this domain but cannot be considered comparable to a well- performed randomized trial) | The methods of outcome assessment were comparable across intervention groups;  and  The outcome measure is only minimally influenced by knowledge of the intervention received by study participants;  and  Any error in measuring the outcome is only minimally related to intervention status. |
| Serious risk of bias (the study has some important problems) | The methods of outcome assessment were not comparable across intervention groups;  or  The outcome measure was subjective (i.e. likely to be influenced by knowledge of the intervention received by study participants) and was assessed by outcome assessors aware of the intervention received by study participants;  or  Error in measuring the outcome was related to intervention status. |
| Critical risk of bias (the study is too problematic to provide any useful evidence on the effects of intervention) | The methods of outcome assessment were so different that they cannot reasonably be compared across intervention groups. |
| No information on which to base a judgement about risk of bias for this domain | No information is reported about the methods of outcome assessment. |

Any of the following means that the study is **at least at moderate risk of bias**:

- The outcome was not clearly and sufficiently described;

Any of the following means that the study is **at least at serious risk of bias**:

- The outcome measure was not valid and reliable;

or

- There were no follow-up outcome measurements conducted at the same time of year as baseline outcome measurements;

or

- (Self-report outcomes only) There was only one follow-up time point;

or

- There were no follow-up outcome measurements conducted sufficiently after completion of the intervention to reduce the ‘novelty effect’;

or

- Participants were aware of being assessed for the purposes of the study

Any of the following means that the study is **at critical risk of bias**:

- The outcome measure was not valid and reliable;

**and**

- Participants were aware of being assessed for the purposes of the study;

**and**

- There were no follow-up outcome measurements conducted at the same time of year as baseline outcome measurements

**The following only apply to systematic observation outcomes:**

Any of the following means that the study is **at least at moderate risk of bias**:

- There was only one follow-up time point;

**and**

- - Outcomes were measured over a period of **more than one week** at each time point

Any of the following means that the study is **at least at serious risk of bias**:

- Outcomes were not measured at multiple times across the course of a day;

or

- Outcomes were not measured across multiple days;

or

- Outcomes were not measured on both weekdays and weekends

or

- There was only one follow-up time point

**and**

- - Outcomes were measured over a period of **one week or less** at each time point

Any of the following means that the study is **at least at critical risk of bias**:

- There was only a single observation period at baseline and follow-up time points;

or

- Follow-up outcome measurements were not conducted at the same time of day as baseline outcome measurements

**7. Bias in selection of the reported result**

| Low risk of bias (the study is comparable to a well-performed randomized trial with regard to this domain) | There is clear evidence (usually through examination of a pre-registered protocol or statistical analysis plan) that all reported results correspond to all intended outcomes, analyses and sub-cohorts. |
| --- | --- |
| Moderate risk of bias (the study is sound for a non-randomized study with regard to this domain but cannot be considered comparable to a well- performed randomized trial) | The outcome measurements and analyses are consistent with an a priori plan; or are clearly defined, and internally and externally consistent;  and  There is no indication of selection of the reported analysis from among multiple analyses;  and  There is no indication of selection of the cohort or subgroups for analysis and reporting on the basis of the results. |
| Serious risk of bias (the study has some important problems) | Outcome measurements or analyses are internally or externally inconsistent;  or  There is a high risk of selective reporting from among multiple analyses;  or  The cohort or subgroup is selected from a larger study for analysis and appears to be reported on the basis of the results. |
| Critical risk of bias (the study is too problematic to provide any useful evidence on the effects of intervention) | There is evidence or strong suspicion of selective reporting of results, and the unreported results are likely to be substantially different from the reported results. |
| No information on which to base a judgement about risk of bias for this domain | There is too little information to make a judgement, for example if only an abstract is available for the study. |

Any of the following means that the study is **at least at serious risk of bias**:

- There was no study protocol published and the authors did not provide a clear and compelling justification for not publishing a study protocol;

Any of the following means that the study is **at least at critical risk of bias**:

- All of the study’s pre-specified analysis and outcomes were **not** conducted and reported in the pre-specified way as stated in the study protocol; and there was no compelling justification for not doing so

**Overall risk of bias**

| **Overall risk of bias judgement for the outcome** | **Criteria (based on the seven risk of bias domains)** |
| --- | --- |
| Low risk of bias (the study is comparable to a well-performed randomized trial with regard to this domain) | The study is judged to be at **low risk of bias for all domains.**  *N.B. Because it will be rare that a non-randomised study is judged as at low risk of bias due to confounding, it is anticipated that most non-randomised studies will be judged as* ***at least*** *at moderate overall risk of bias* |
| Moderate risk of bias (the study is sound for a non-randomized study with regard to this domain but cannot be considered comparable to a well- performed randomized trial) | The study is judged to be at **low or moderate risk of bias for all domains.** |
| Serious risk of bias (the study has some important problems) | The study is judged to be at **serious risk of bias** in at least one domain, but not at critical risk of bias in any domain. |
| Critical risk of bias (the study is too problematic to provide any useful evidence on the effects of intervention) | The study is judged to be at **critical risk of bias in at least one domain.** |
| No information on which to base a judgement about risk of bias for this domain | There is no clear indication that the study is at serious or critical risk of bias and there is a lack of information in one or more key domains of bias *(a judgement is required for this).* |

The mapping of domain-level judgements to overall judgements described in this table is a programmable algorithm. However, in practice some ‘Serious’ risks of bias (or ‘Moderate’ risks of bias) might be considered to be additive, so that ‘Serious’ risks of bias in multiple domains can lead to an overall judgement of ‘Critical’ risk of bias (and, similarly, ‘Moderate’ risks of bias in multiple domains can lead to an overall judgement of ‘Serious’ risk of bias). Therefore, if an outcome has **four or more** judgements of ‘Moderate’ or ‘Serious’ risks of bias then this leads to an overall risk of bias judgement of ‘Serious’ or ‘Critical’ respectively e.g., if an outcome is at ‘Serious’ risk of bias in four domains, then this outcome has an overall ‘Critical’ risk of bias.
