# Supplementary material for: The effect of changing the built environment on physical activity: a quantitative review of the risk of bias in natural experiments
Source: Int J Behav Nutr Phys Act. 2016 Oct 7;13:107. doi: 10.1186/s12966-016-0433-3 (PMC5055702; doi:10.1186/s12966-016-0433-3)
Supplement: Additional file 4: — The adapted ACROBAT-NRSI used for the present review. (DOCX 37 kb) [file 12966_2016_433_MOESM4_ESM.docx]

**Additional file 4. The adapted ACROBAT-NRSI used for the present review**

**Study characteristics**

Author (year)

|  |
| --- |

Study design

|  |
| --- |

Study location

|  |
| --- |

Type of intervention (cost)

|  |
| --- |

List any explicitly reported co-interventions that could differ between intervention groups and could have an impact on study outcomes

|  |
| --- |

Number of control sites

|  |
| --- |

Physical activity outcomes

|  |
| --- |

Timing of intervention

|  |
| --- |

Timing of outcome measurements

|  |
| --- |

Sample size

|  |
| --- |

Results

|  |
| --- |

**Risk of bias assessment**

List of abbreviations

- NA: Not Applicable
- Y: Yes
- PY: Probably Yes
- PN: Probably No
- N: No
- NI: No Information

**Important notes**

- In studies with multiple outcomes, separate risk of bias assessments must be conducted for each outcome.
- The questions have been worded so that all Y or PY answers indicate a **lower risk of bias**, whereas N or PN answers indicate a **higher risk of bias**.
- It is essential that the reasons are provided for any judgements of ‘serious’ or ‘critical’ risk of bias.
- Declaring a study to be at a particular level of risk of bias for an individual domain will mean that the study as a whole has a risk of bias at least this severe (for the outcome being assessed). For example, a judgement of ‘Serious risk of bias’ within any domain should have similar implications for the study as a whole irrespective of which domain is being assessed.
- The phrase ‘time point’ refers to the interval at which measurements were taken (e.g., baseline, first follow-up, second follow-up etc.), whereas the phrase ‘observation period’ refers to each measurement period for systematic observations (e.g., 10:00am-12:00am, 1:00pm-3:00pm, 7:00pm-9:00pm etc.).

| Bias due to confounding | 1.1. Is confounding of the effect of intervention unlikely in this study?  *N.B. This is likely to be N or PN for most studies in this area since it is rare that a non-randomised study is judged as being at low risk of bias due to confounding*  **If Y or PY to 1.1:** the study can be considered to be at low risk of bias due to confounding and no further signalling questions need be considered | NA / Y / PY / PN / N / NI | (Description) |
| --- | --- | --- | --- |
|  | **If N or PN to 1.1:** |  |  |
|  | 1.2. Were participants analysed according to their initial intervention group throughout follow up?  *N.B. If participants could switch between intervention and control groups then associations between intervention and outcome may be biased by time-varying confounding. This occurs when prognostic factors influence switches between intended interventions*  *N.B. For systematic observations, assessors should consider whether it is likely that participants could be double counted in intervention and controls groups based on the distance between intervention and control sites*  **If Y or PY to 1.2**, answer questions 1.4 to 1.10, which relate to baseline confounding | NA / Y / PY / PN / N / NI | (Description) |
|  | 1.3. **If N or PN to 1.2**: Were intervention discontinuations or switches unlikely to be related to factors that are prognostic for the outcome?  *N.B. Only answer N or PN if it is likely that intervention discontinuations or switching between intervention and control groups will significantly impact upon the outcome e.g., only a handful of participants relocating out of their intervention or control group, or switching between intervention and control groups, is less likely to significantly bias the outcome* | NA / Y / PY / PN / N / NI | (Description) |
|  | 1.4. Did the authors use an appropriate analysis method that adjusted for all the critically important confounding domains? Consider whether the authors controlled for…  *N.B. Answer Y or PY for questions 1.5 to 1.9 if the confounding variable was similar across intervention and control groups*  *N.B. Only answer NA if the confounding variable is judged as irrelevant for the outcome* | NA / Y / PY / PN / N / NI | (Description) |
|  | 1.5. … differences in baseline outcome measurements?  *N.B. If no statistical analysis was conducted to test for significant differences in outcome measurements at baseline then a judgement should be made based on the available data*  *N.B. If there is no information to make a judgement then answer PN* | NA / Y / PY / PN / N / NI | (Description) |
|  | 1.6. … differences in baseline demographic characteristics?  *N.B. To answer Y or PY, the study must consider age* ***and*** *gender*  *N.B. If no statistical analysis was conducted to test for significant differences in demographic characteristics at baseline then a judgement should be made based on the available data*  *N.B. This needs to include the characteristics of individual participants from the sample rather than neighbourhood-level characteristics*  *N.B. If there is no information to make a judgement then answer PN* | NA / Y / PY / PN / N / NI | (Description) |
|  | 1.7. … any unusual events? (E.g., cultural or religious events, sporting events, music festivals etc.)  *N.B. If there is no reference to any unusual events then answer NI* | NA / Y / PY / PN / N / NI | (Description) |
|  | 1.8. … socioeconomic or political influences? (E.g. natural disasters, crime and conflict etc.)  *N.B. If there is no reference to any socioeconomic or political influences then answer NI* | NA / Y / PY / PN / N / NI | (Description) |
|  | 1.9. **If Y or PY to 1.4**: Were confounding domains that were adjusted for measured validly and reliably by the variables available in this study? | NA / Y / PY / PN / N / NI | (Description) |
|  | 1.10. Did the authors avoid adjusting for post-intervention variables?  *N.B. This relates to adjusting for mediating variables, which is likely to be Y or PY for most studies in this area because of the lack of clarity on the causal mechanisms underlying the relationship between the built environment and physical activity* | NA / Y / PY / PN / N / NI | (Description) |
|  | **If N or PN to 1.2 and 1.3:** |  |  |
|  | 1.11. Did the authors use an appropriate analysis method that adjusted for all the critically important confounding domains and for time-varying confounding?  *N.B. This is likely to be NA for most studies in this area since interventions are relatively permanent and tend not to change over time* | NA / Y / PY / PN / N / NI | (Description) |
|  | 1.12. **If Y or PY to 1.11**: Were confounding domains that were adjusted for measured validly and reliably by the variables available in this study? | NA / Y / PY / PN / N / NI | (Description) |
|  | Questions 1.13 and 1.14 are only applicable for outcomes using systematic observation or live data collected within a specified period of time (e.g., accelerometers) |  |  |
|  | 1.13. Were weather conditions similar across all observation periods?  *N.B. Answer Y or PY if the outcome used a validated metholdology and weather conditions are accounted for in the methodology protocol e.g., with SOPARC observations are to be made during clement weather conditions*  *N.B. To answer Y or PY, the study must consider precipitation* ***and*** *temperature*  *N.B. Answer NI if there is no reference to the weather conditions* | NA / Y / PY / PN / N / NI | (Description) |
|  | 1.14. **If N or PN to 1.13**: Did the authors use an appropriate analysis method that adjusted for differences in weather conditions? | NA / Y / PY / PN / N / NI | (Description) |
|  | 1.15. Did the authors attempt to match the control site with the intervention site? | NA / Y / PY / PN / N / NI | (Description) |
|  | **If Y or PY to 1.15:** |  |  |
|  | 1.16. How did they match the control site with the intervention site? | - | (Description) |
|  | 1.17. Were the intervention and control site matched using any variables based on features of the built environment?  *N.B. This includes any aspect of the built environment e.g., land use, population density, street connectivity, physical infrastructure* | NA / Y / PY / PN / N / NI | (Description) |
|  | 1.18. Were the intervention and control site matched using any variables based on demographics? | NA / Y / PY / PN / N / NI | (Description) |
|  | 1.19. Is the control site well matched to the intervention site?  *N.B. Given the heterogeneity of built environment interventions, assessors should make a judgement on a study-by-study basis. This is less likely to be judged as Y or PY if 1.18 or 1.19 have been judged as N or PN* | NA / Y / PY / PN / N / NI | (Description) |
|  | **If N or PN to 1.19:** |  |  |
|  | 1.20. Did the authors use an appropriate analysis method that adjusted for all the critically important differences between control and intervention sites? | NA / Y / PY / PN / N / NI | (Description) |
|  | 1.21. Were there multiple control sites?  *N.B. Pooling data from separate control sites is classed as using multiple control sites* | NA / Y / PY / PN / N / NI | (Description) |
|  | 1.22. Is it unlikely that the control site underwent any significant changes during the study period that did not similarly occur in the intervention site and could influence the outcome?  *N.B. Assessors should consider whether these changes were large enough to significantly influence the outcome* | NA / Y / PY / PN / N / NI | (Description) |
|  | 1.23. **If N or PN to 1.22:** Did the authors use an appropriate analysis method that adjusted for these significant changes? | NA / Y / PY / PN / N / NI | (Description) |
|  | **Risk of bias judgement** | Low / Moderate / Serious / Critical / NI | (Support for judgement) |
|  | Optional: What is the predicted direction of bias due to confounding? | Favours experimental / Favours comparator / Towards null /Away from null / Unpredictable | (Rationale) |
| Bias in selection of participants into the study | - 1. Is there a fully justified sample size calculation? | NA / Y / PY / PN / N / NI | (Description) |
|  | - 1. Are the sampling criteria for participants clearly described?   *N.B. For systematic observation outcomes, this should include a clear definition of the types of physical activity being measured (e.g., sedentary, moderate, vigorous), as well as the days and times of the week when outcome measurements are taken* | NA / Y / PY / PN / N / NI | (Description) |
|  | - 1. Is there a clear and sufficient description of the sample?   *N.B. To answer Y or PY, the study must include age* ***and*** *gender. This needs to include the characteristics of individual participants from the sample rather than neighbourhood-level characteristics*  *N.B. To answer Y or PY, it should be possible to judge whether characteristics of control and intervention participants differ e.g., it may not always be possible to judge whether characteristics of control and intervention participants differ if only medians are reported*  *N.B. This can be judged as NA for outcomes where the authors have appropriately controlled for differences between intervention and control groups e.g., using propensity score analysis* | NA / Y / PY / PN / N / NI | (Description) |
|  | - 1. Was selection into the study unrelated to intervention?   *N.B. This refers to when selection is related to an effect of either intervention or a cause of intervention (self-selection) e.g., whether selected participants have recently relocated because of this intervention*  *N.B. This is likely to be NI for most studies in this area, particularly population-level outcomes, due to limited information on participants* | NA / Y / PY / PN / N / NI | (Description) |
|  | - 1. Was selection into the study unrelated to outcome?   *N.B. This refers to when selection is related to an effect of either the outcome or a cause of the outcome e.g., whether selected participants are more inclined to be physically active*  *N.B. This is likely to be NI for most studies in this area, particularly population-level outcomes, due to limited information on participants* | NA / Y / PY / PN / N / NI | (Description) |
|  | - 1. Do start of follow-up and start of intervention coincide for most subjects?   *N.B. This relates to whether follow-up was conducted as soon as intervention construction was completed*  *N.B. This is likely to be N or PN for most studies in this area since it is rare that authors will measure physical activity levels immediately after completion of the intervention to avoid capturing the ‘novelty effect’* | NA / Y / PY / PN / N / NI | (Description) |
|  | 2.7. **If N or PN to 2.4 or 2.5 or 2.6**: Were adjustment techniques used that are likely to correct for the presence of selection biases? | NA / Y / PY / PN / N / NI | (Description) |
|  | **Risk of bias judgement** | Low / Moderate / Serious / Critical / NI | (Support for judgement) |
|  | Optional: What is the predicted direction of bias due to selection of participants into the study? | Favours experimental / Favours comparator / Towards null /Away from null / Unpredictable | (Rationale) |
| Bias in measurement of interventions | Questions 3.1 and 3.2 are only applicable to studies that **did** **not** sample from the whole intervention site |  |  |
|  | 3.1. Was the sampling site selected using probability-based sampling? | NA/ Y / PY / PN / N / NI | (Description) |
|  | 3.2. **If N or PN to 3.1**: Was the selection of the sampling site appropriately justified to capture a valid representation of the whole intervention? | NA / Y / PY / PN / N / NI | (Description) |
|  | Did the authors describe… |  |  |
|  | 3.3. … what was modified in the intervention?  *N.B. The study should at least report the type of intervention and the length or size of the intervention* | NA / Y / PY / PN / N / NI | (Description) |
|  | 3.4. … where the intervention was implemented?  *N.B. Assessors should consider whether the intervention could be roughly located on a map based on the information provided* | NA / Y / PY / PN / N / NI | (Description) |
|  | 3.5. … how long it took to construct the intervention?  *N.B. Assessors should be able to roughly determine when intervention construction started and finished* | NA / Y / PY / PN / N / NI | (Description) |
|  | 3.6. **If N or PN to 3.5:** Is it unlikely that intervention construction could overlap with outcome measurements? | NA / Y / PY / PN / N / NI | (Description) |
|  | Questions 3.7 to 3.9 are **not** applicable for systematic observation outcomes |  |  |
|  | 3.7. Is intervention status well defined? | NA / Y / PY / PN / N / NI | (Description) |
|  | 3.8. Was information on intervention status recorded at the time of intervention? | NA / Y / PY / PN / N / NI | (Description) |
|  | 3.9. Was information on intervention status unaffected by knowledge of the outcome or risk of the outcome?  *N.B. This relates to whether the definition of intervention status could be influenced by knowledge or likelihood of the outcome* | NA / Y / PY / PN / N / NI | (Description) |
|  | **Risk of bias judgement** | Low / Moderate / Serious / Critical / NI | (Support for judgement) |
|  | Optional: What is the predicted direction of bias due to measurement of outcomes or interventions? | Favours experimental / Favours comparator / Towards null /Away from null / Unpredictable | (Rationale) |
| Bias due to departures from intended interventions | Questions 4.1 and 4.2 are only applicable for studies that have **explicitly reported** any other physical activity interventions that occurred during the study period aside from the built environment interventions under investigation |  |  |
|  | 4.1. Were the critical co-interventions balanced across intervention groups? | NA / Y / PY / PN / N / NI | (Description) |
|  | 4.2. Were numbers of switches to other interventions low? | NA / Y / PY / PN / N / NI | (Description) |
|  | 4.3. Is it unlikely that any delays or changes in intervention construction impacted upon the study? | NA / Y / PY / PN / N / NI | (Description) |
|  | 4.4. **If N or PN to 4.3**: Were adjustment techniques used that are likely to correct for these issues? | NA / Y / PY / PN / N / NI | (Description) |
|  | Questions 4.5 and 4.6 are **not** applicable for population-level outcomes or any outcomes measured directly from the intervention site (e.g., intercept surveys) |  |  |
|  | 4.5. Was individual-level intervention exposure measured?  *N.B. This refers to whether authors attempted to measure participants’ actual exposure to the built environment intervention. This will enable the authors to determine the extent to which changes in the outcome are specifically attributable to exposure to the intervention* | NA / Y / PY / PN / N / NI | (Description) |
|  | 4.6. **If Y or PY to 4.5**: Was individual-level intervention exposure measured objectively? | NA / Y / PY / PN / N / NI | (Description) |
|  | **Risk of bias judgement** | Low / Moderate / Serious / Critical / NI | (Support for judgement) |
|  | Optional: What is the predicted direction of bias due to departures from the intended interventions? | Favours experimental / Favours comparator / Towards null /Away from null / Unpredictable | (Rationale) |
| Bias due to missing data | 5.1. Are outcome data reasonably complete?  *N.B. This aims to elicit whether the proportion of missing observations is likely to result in missing information that could substantially impact on our ability to answer the question being addressed.*  *N.B. Although no exact single threshold can determine the judgement for response rates in all studies, a response rate of 70% is provided as an approximate figure to help judge completeness of outcome data*  *N.B. For systematic observations, consider whether it is likely that physical activity behaviours were missed e.g., busy observation periods increases the likelihood of missing data unless reliability of agreement between observers was high* | NA / Y / PY / PN / N / NI | (Description) |
|  | 5.1a. What was the initial response rate of participants eligible at baseline? | - | (Description) |
|  | 5.1b. What was the response rate of participants sampled at follow-up? | - | (Description) |
|  | 5.1c. What was the overall response rate?  *N.B. For repeated cross-sectional outcomes, overall response rates are defined as the average of all response rates at each time point. For within-person longitudinal outcomes, overall response rates are defined as participants from the initial sample who completed surveys at all time points.* | - | (Description) |
|  | Question 5.2 is **not** applicable for systematic observation outcomes |  |  |
|  | 5.2. Was intervention status reasonably complete for those in whom it was sought? | NA / Y / PY / PN / N / NI | (Description) |
|  | 5.3. Are data reasonably complete for other variables in the analysis?  *N.B. This question relates particularly to participants excluded from the analysis because of missing information on confounders that were adjusted for in the analysis* | NA / Y / PY / PN / N / NI | (Description) |
|  | **If N or PN to 5.1, 5.2 or 5.3** |  |  |
|  | 5.4. Are the proportion of participants and reasons for missing data similar across interventions? | NA / Y / PY / PN / N / NI | (Description) |
|  | 5.5. Were appropriate statistical methods used to account for missing data? | NA / Y / PY / PN / N / NI | (Description) |
|  | **Risk of bias judgement** | Low / Moderate / Serious / Critical / NI | (Support for judgement) |
|  | Optional: What is the predicted direction of bias due to missing data? | Favours experimental / Favours comparator / Towards null /Away from null / Unpredictable | (Rationale) |
| Bias in measurement of outcomes | 6.1. Was the outcome clearly and sufficiently described?  *N.B. Assessors should consider whether it would be possible to replicate this outcome using the information provided*  *N.B. This should include the types of physical activity being measured (e.g., sedentary, moderate, vigorous)* | NA / Y / PY / PN / N / NI | (Description) |
|  | 6.2. Was the outcome measure valid and reliable?  *N.B. Assessors should consider whether validity and reliability assessments are necessary e.g., simple counts of cyclists is likely to be a valid and reliable outcome measure. Otherwise, unless authors have used a widely established, validated outcome measure such as SOPARC methodology, authors need to have reported the validity and reliability of their outcome measure* | NA / Y / PY / PN / N / NI | (Description) |
|  | 6.3. Was the outcome measure objective?  *N.B. Systematic observations are classed as objective* | NA / Y / PY / PN / N / NI | (Description) |
|  | Questions 6.4 to 6.9 are only applicable for outcomes using systematic observation or live data collected within a specified period of time (e.g., accelerometers) |  |  |
|  | 6.4. Were there multiple baseline and follow-up observation periods?  *N.B. This is likely to be Y or PY for most studies in this area since it is rare that a study will only observe physical activity during a single observation period at baseline and follow-up* | NA / Y / PY / PN / N / NI | (Description) |
|  | **If Y or PY to 6.4:** |  |  |
|  | 6.5. Were the outcomes measured at multiple times across the course of a day? | NA / Y / PY / PN / N / NI | (Description) |
|  | 6.6. Were the outcomes measured across multiple days? | NA / Y / PY / PN / N / NI | (Description) |
|  | 6.7. Were the outcomes measured on both weekdays and weekends? | NA / Y / PY / PN / N / NI | (Description) |
|  | 6.8. Were the outcomes measured over a period of more than one week at each time point? | NA / Y / PY / PN / N / NI | (Description) |
|  | 6.9. Were follow-up outcome measurements conducted at the same time of day as baseline outcome measurements? | NA / Y / PY / PN / N / NI | (Description) |
|  | 6.10. Were any follow-up outcome measurements conducted at the same time of year as baseline outcome measurements? | NA / Y / PY / PN / N / NI | (Description) |
|  | 6.11. Were there multiple follow-up time points? | NA / Y / PY / PN / N / NI | (Description) |
|  | 6.12. Were any follow-up outcome measurements conducted sufficiently after completion of the intervention to reduce the ‘novelty effect’?  *N.B. It is generally accepted that follow-up outcome measurements conducted at least 12 months after completion of the intervention is sufficient, but given the heterogeneity of built environment interventions, assessors should make a judgement on a study-by-study basis.* | NA / Y / PY / PN / N / NI | (Description) |
|  | 6.13. Were participants unaware of being assessed for the purposes of the study?  *N.B. To answer Y or PY, participants were aware of being assessed* ***and*** *knew the purposes of the study* | NA / Y / PY / PN / N / NI | (Description) |
|  | 6.14. Were outcome assessors unaware of the intervention received by study participants?  *N.B. In studies where participants report their outcomes themselves, for example in a questionnaire, the outcome assessor is the study participant* | NA / Y / PY / PN / N / NI | (Description) |
|  | 6.15. Were the methods of outcome assessment comparable across intervention groups?  *N.B. Comparable assessment methods (i.e. data collection) would involve the same outcome detection methods and thresholds, same time point (especially for systematic observation), same definition and same measurements* | NA / Y / PY / PN / N / NI | (Description) |
|  | 6.16. Were any systematic errors in measurement of the outcome unrelated to intervention received?  *N.B. This will usually be due either to outcome assessors being aware of the intervention received or to non-comparability of outcome assessment methods* | NA / Y / PY / PN / N / NI | (Description) |
|  | **Risk of bias judgement** | Low / Moderate / Serious / Critical / NI | (Support for judgement) |
|  | Optional: What is the predicted direction of bias due to measurement of outcomes? | Favours experimental / Favours comparator / Towards null /Away from null / Unpredictable | (Rationale) |
| Bias in selection of the reported result | 7.1. Was a study protocol published? | NA / Y / PY / PN / N / NI | (Description) |
|  | 7.2. **If N or PN to 7.1**: Did the authors provide a clear and compelling justification for not publishing a study protocol? | NA / Y / PY / PN / N / NI | (Description) |
|  | 7.3. **If Y or PY to 7.1**: Are all of the study’s pre-specified analysis and outcomes conducted and reported in the pre-specified way? | NA / Y / PY / PN / N / NI | (Description) |
|  | 7.4 **If N or PN to 7.3**: Did the authors provide a clear and compelling justification for not conducting and reporting the study’s pre-specified analysis and outcomes in the pre-specified way? | NA / Y / PY / PN / N / NI | (Description) |
|  | Is the reported effect estimate unlikely to be selected, on the basis of the results, from... |  |  |
|  | 7.5. ... multiple outcome measurements within the outcome domain? | NA / Y / PY / PN / N / NI | (Description) |
|  | 7.6 ... multiple analyses of the intervention-outcome relationship? | NA / Y / PY / PN / N / NI | (Description) |
|  | 7.7 ... different subgroups? | NA / Y / PY / PN / N / NI | (Description) |
|  | **Risk of bias judgement** | Low / Moderate / Serious / Critical / NI | (Support for judgement) |
|  | Optional: What is the predicted direction of bias due to measurement of outcomes? | Favours experimental / Favours comparator / Towards null /Away from null / Unpredictable | (Rationale) |
| Overall bias | **Risk of bias judgement**  *N.B. If an outcome has* ***four or more*** *judgements of ‘Moderate’ or ‘Serious’ risks of bias then this leads to an overall risk of bias judgement of ‘Serious’ or ‘Critical’ respectively* | Low / Moderate / Serious / Critical / NI | (Support for judgement) |
|  | Optional: What is the predicted direction of bias for this outcome? | Favours experimental / Favours comparator / Towards null /Away from null / Unpredictable | (Rationale) |
