# Supplementary material for: The effect of changing the built environment on physical activity: a quantitative review of the risk of bias in natural experiments
Source: Int J Behav Nutr Phys Act. 2016 Oct 7;13:107. doi: 10.1186/s12966-016-0433-3 (PMC5055702; doi:10.1186/s12966-016-0433-3)
Supplement: Additional file 5: — Detailed summary of the key characteristics and results of included studies. (DOCX 29 kb) [file 12966_2016_433_MOESM5_ESM.docx]

**Additional file 5. Detailed summary of the key characteristics and results of included studies**

| **Author** | **Study location** | **Research design** | **Type of intervention (total cost)** | **Physical activity outcomes** | **Timing of intervention** | **Timing of outcome measurements** | **Sample size** | **Number of control sites** | **Results** |
| --- | --- | --- | --- | --- | --- | --- | --- | --- | --- |
| Branas *et al*. (79) | Philadelphia, US | Repeated cross-sectional | Greening of 4,436 abandoned vacant lots over 725,000m^2^  (cost not reported) | Survey (Southeastern Pennsylvania Household Health Survey) included a self-report question of physical activity levels | 1999 to 2008 | Used waves from the survey every two years from 1998 to 2008 | No exact count provided | 13,308 matched control lots were randomly selected and assigned at a ratio of three control lots per treated lot | +ve  Greening was associated with residents’ reporting more exercise (p < .01) |
| Cohen *et al*. (80) | Southern California, US | Mixed: repeated cross-sectional and within-person longitudinal | 5 parks, ranging from 3.4 to 16 acres, underwent major improvements (Over $1 million budget per park) | 1) SOPARC: 4 observation periods per day for 7 days  2) Self-reported park use and physical activity using household interviews with residents within 2 miles from each park  3) Self-reported park use and physical activity using intercept interviews | Unclear | Baseline: December 2003 to November 2004;  Follow-up: April 2006 to March 2008 | 1) 3,500 park users  2) 1,480 park users  3) 1,387 household residents | 5 matched control parks, one per intervention site | -ve  Overall park use and physical activity declined in both intervention and control parks |
| Cohen *et al*. (81) | Los Angeles, US | Repeated cross-sectional | A skate park ($3.5 million) and a senior citizen’s centre ($3.3 million) underwent major renovations | SOPARC: 4 observation periods per day for 7 days | Unclear (The skate park and senior citizen’s centre were both closed for 2 years during renovation) | Unclear (follow-up conducted 1 to 3 months following re-opening of renovated areas) | Senior centre: 2,188 users  Skate park: no exact count provided | 1 control site per intervention; one skate park and one senior centre | Mixed  +ve  510% increase in skate park use compared to 77% in control skate park  -ve  The senior centre had substantially fewer users |
| Cohen *et al*. (82) | Los Angeles, US | Repeated cross-sectional | 12 parks, ranging from 0.5 to 46 acres, had “Family Fitness” Zones (outdoor gyms) installed (average of $45,000 for each park) | 1) SOPARC: 3 observation periods per day for 4 days  2) Self-reported park use and use of fitness equipment using intercept interviews with park users | Unclear | Baseline: Winter 2008 to 2009;  Follow-up 1: Winter 2008 to 2009;  Follow-up 2: Spring 2010  (the timing of data collection from baseline to follow-up in the control parks was an average of 2 years compared to 1 year in the intervention parks) | 1) 9,476 park users  2) 2,636 interviews | 10 matched control parks that did not install Fitness Zones | Mixed  +ve  Self-reports of being a new park user increased more in Fitness Zones parks and estimated energy expenditure in Fitness Zones parks was higher at both follow-ups than at baseline  -ve  The number of park users increased by 11% compared to control parks but this difference was not statistically significant |
| Fitzhugh *et al*. (83) | Knoxville, US | Repeated cross-sectional | An urban greenway/trail 2.9 miles long and 8 foot wide was retrofıtted in a neighbourhood that lacked connectivity of the residential pedestrian infrastructure to non-residential destinations ($2.1 million) | Systematic observations: in the general neighbourhood, 3 observation periods per day for 2 days over one week.  At the school level to measure active transport to school, 2 observation periods per day for 2 days over one week | May 2005 to December 2005 | Baseline: March 2005; Follow-up: March 2007 | Exact counts not provided | 2 matched control neighbourhoods | +ve  Physical activity significantly increased between 2005 and 2007 (p = 0.000) in the intervention neighbourhood. The control neighbourhoods had a significant decrease in physical activity (p = 0.000).  The pre- and post-intervention changes between experimental and control neighbourhoods were significantly different for total physical activity (p = 0.001); walking (p = 0.001); and cycling (p = 0.038). There was no significant change over time for active transport to school |
| Gustat *et al*. (84) | New Orleans, US | Repeated cross-sectional | A 6-block walking path was built and a school playground was installed in a neighbourhood (cost not reported) | 1) Self-reported physical activity using door-to-door surveys  2) SOPLAY: 1 observation period over 3 days a week, for a 6-week period | Path: November 2007  Playground: May 2007 | 1) Baseline: September 2006 to February 2007; Follow-up: October 2008 to January 2009  2) Baseline: October to December 2006; Follow-up: October to December 2008 | 1) 1,191 interviews  2) No exact count provided | 2 matched comparison neighbourhoods | +ve  The proportion of residents observed who were active increased significantly in the section of the intervention neighbourhood with the path compared with comparison neighbourhoods |
| Krizek *et al*. (85) | Minneapolis / St. Paul, US | Repeated cross-sectional | Installation of bicycle lanes and off-street bicycle paths (cost not reported) | Census data: proportion of bicycle commuters against total number of commuters | Completed during the 1990s | Baseline: 1990; Follow-up: 2000 | Exact count not provided | 1 buffer zone: based on distance from intervention facilities | +ve  Areas closer to new bicycle facilities showed more of an increase in bicycle mode share than areas further away, although all areas had increases |
| Merom *et al*. (86) | Sydney, Australia | Mixed: repeated cross-sectional and within-person longitudinal | Construction of a Rail Trail and a local promotional campaign to develop community awareness of the facility (cost not reported) | 1) Self-reported walking and cycling behaviour using population-based telephone surveys  2) Objective concurrent monitoring of daily bike counts using 24 hour bike counters at 4 locations along the Rail Trail | Rail Trail: unclear when construction started, finished on 2^nd^ December 2000;  Promotional campaign: 2^nd^ December 2000 to 29^th^ February 2001 | 1) Baseline: November 16^th^ to December 4^th^ 2000; Follow-up: March 1^st^ to March 20^th^ 2001  2) Baseline: 20^th^ October 2000 to 2^nd^ December 2000; Follow-up: 3^rd^ December 2000 to 15^th^ March 2001 | 1) 450 households at follow-up  2) No exact count provided | 1 outer area: random sample of bicycle-owner households (aged 18 to 55) located 1.5 to 5 km from the Rail Trail | +ve  Trail usage was higher among bike-owners than pedestrians (8.9 vs 3.3%, p = 0.014) and was moderated by proximity to the Rail Trail. Inner cyclists increased mean cycling time while outer cyclists decreased cycling time. Mean daily bike counts in the monitored areas increased significantly after the Rail Trail launch in the two suburbs (p = 0.0001, and p = 0.0004) |
| Parker *et al*. (87) | New Orleans, US | Repeated cross-sectional | A 1-mile, 5-foot wide dedicated bike lane was striped on both sides of the road (cost not reported) | Systematic observation of cyclists | June 2010 | Baseline: September 2009; Follow-up: September 2010 | Exact count provided | 2 adjacent streets | +ve  More people rode in the overall neighbourhood after the lanes were striped; however, the increase in cyclists was greatest on the street with the new bike lane |
| Tester and Baker (88) | San Francisco, US | Repeated cross-sectional | 2 public parks in low-income neighbourhoods underwent playfield renovations and training and skills development for park and recreation program staff  ($5.5 million) | SOPARC: 8 observation periods per day for 7 consecutive days | Summer of 2006 | Baseline: 30^th^ May to 5^th^ June 2006; Follow-up: 30^th^ May to 5^th^ June 2007 | 4,889 park visitors | 1 matched control park | +ve  Both intervention park playfields saw significant increases in male and female visitors, with over a 4-fold increase in the average number of visitors per observation among most age groups. For both genders, there was a significant increase in sedentary, moderately active, and vigorously active visitors to the intervention park playfields |
| Veitch *et al*. (89) | Victoria, Australia | Repeated cross-sectional | A park (size: 25,200 m^2^) was refurbished; including the establishment of a fenced leash-free area for dogs (12,800 m^2^); an all-abilities playground; a 365-metre walking track; a barbecue area; landscaping; and fencing, to prevent motor vehicle access to the park (cost not reported) | SOPARC: 3 observation periods per day for 9 days over 4 weeks | November to December 2009 | Baseline: August to September 2009; Follow-up 1: March to April 2010; Follow-up 2: August to September 2010 | 2,050 park users | 1 matched control park (size: 10,000 m^2^) | +ve  In the intervention park, there were significant increases from pre- to post-improvement in the number of park users and the number of people observed walking and being vigorously active. At the control park, there were no significant differences in walking and vigorous activity among the three time points |
| West and Shores (90) | Roanoke, US | Within-person longitudinal | 5 miles of greenway were added to an existing greenway (cost not reported) | Self-reported physical activity: days in the past week spent walking, participating in moderate or vigorous physical activity | Early 2008 | Baseline: December 2007; Follow-up: December 2008 (11 months post-intervention) | 166 residents | 1 buffer zone: residents living further away from the greenway (within 0.51 to 1.0 mile radius of the greenway), in comparison to the intervention group defined as those living within a 0.5 mile radius of the greenway | -ve  Despite overall increases in days spent walking and participating in moderate physical activity in all participants, there was no significant difference between the intervention and control group |
