# Supplementary material for: The effect of changing the built environment on physical activity: a quantitative review of the risk of bias in natural experiments
Source: Int J Behav Nutr Phys Act. 2016 Oct 7;13:107. doi: 10.1186/s12966-016-0433-3 (PMC5055702; doi:10.1186/s12966-016-0433-3)
Supplement: Additional file 6: — Summary of suggestions to improve future research in this area. (DOCX 26 kb) [file 12966_2016_433_MOESM6_ESM.docx]

**Additional file 6. Summary of suggestions to improve future research in this area**

| **Research priority** | **Suggestions for future research** |
| --- | --- |
| **1. Better matching of control sites and more nuanced use of graded exposure** | **What can be done now?**   - Studies must include a control/comparison group. - When using a parallel-group design with ‘exposed’ and ‘unexposed’ comparison groups, control sites should be matched using objective features of the built environment and demographic characteristics. Studies should also clearly report how control sites were matched with intervention sites.   **What needs further investigation?**   - Investigate the built environmental variables that control and intervention sites should be matched upon. - Explore the practicalities of identifying control sites located sufficiently far away from intervention sites to reduce the risk of contamination. - More research into developing specific distance-based intervention and comparison groups that take into account differences in exposure between individuals who reside within the same geographical area. |
| **2. Use of multiple control sites** | **What can be done now?**   - Use multiple control sites (including different types of control sites e.g., pre-intervention condition, matched control, synthetic control).   **What needs further investigation?**  **-** |
| **3. Controlling for confounding domains** | **What can be done now?**   - Statistically test for baseline differences between intervention and control groups, particularly demographic characteristics and outcome measurements. For demographic characteristics, future research should consider age and gender as these characteristics are consistently correlated with physical activity [35]. - Control for any baseline differences between intervention and control groups using appropriate methods, as recommended by MRC guidelines for natural experiments [11]. - Check for unusual events and socioeconomic or political influences during the study period, and attempt to control for the presence of any of these confounding variables.   **What needs further investigation?**  - |
| **4. Publishing study protocols with a priori analyses specified** | **What can be done now?**   - Publish pre-registered study protocols detailing the design, procedures and analysis that will be used in the study.   **What needs further investigation?**   - Development of standards that detail what should be included in a study protocol for research in this area. - Establish closer partnerships between researchers, research funders, practitioners, local governments and community-based organisations to improve flexibility and rigour in evaluations of built environment interventions. |
| **5. Use of adequate outcome measurements** | **What can be done now?**   - Studies should not rely solely on self-report measures of physical activity. - Triangulation between observational measures and self-report or accelerometer data to provide reassurance that findings are robust to the different types of bias associated with each individual method of measurement. - When using systematic observation, conduct inter-rater reliability assessments between observers and report this accordingly. Also, observations should be conducted over a period of more than one week at each time-point. - Conduct multiple follow-up time points. - Carry out follow-up outcome measurements at least 12 months after completion of the intervention to reduce the ‘novelty effect’ so that ‘normal’ physical activity levels are captured.   **What needs further investigation?**   - Develop validated self-report measures of physical activity. - Develop validated photo- or video-based technology to systematically observe physical activity behaviour. |
| **6. Better reporting of samples and interventions** | **What can be done now?**   - Provide a clear breakdown of outcome data separately for intervention and control groups. At a minimum, this should include age and gender for all groups as these characteristics are consistently correlated with physical activity [35]. - Clearly describe what was modified in the intervention, where it was implemented, and how long it took to construct (including the start and finish date of intervention construction). - It may be helpful, if possible, to provide a map or other photographic information showing where the intervention was located.   **What needs further investigation?**  - |
| **7. Sample size calculations** | **What can be done now?**   - Perform appropriate sample size calculations.   **What needs further investigation?**   - Research the frequencies of people and their physical activity behaviour that occurs in different built environment spaces, on different days and times of the week. This could be facilitated by the development of less costly and less labour-intensive validated photo- or video-based technology to systematically observe physical activity behaviour. |
| **8. Measuring exposure to the intervention at the individual level** | **What can be done now?**   - Individual-level outcomes could objectively measure exposure to the intervention using a combination of GPS monitors and accelerometers (at least in a sub-sample of participants). - Studies should not rely on pre-defined geographic units of physical activity data and ensure that data is collected for the purposes of the study that map onto the built environment of interest.   **What needs further investigation?**   - More research into validated methodology that measures both individual-level intervention exposure and overall physical activity levels. |
